# Supplementary material for: Lanthanide single-atom catalysts for efficient CO2-to-CO electroreduction
Source: Nat Commun. 2025 Mar 27;16:2985. doi: 10.1038/s41467-025-57464-8 (PMC11947204; doi:10.1038/s41467-025-57464-8)
Supplement: Supplementary file 1 — Supplementary Information [file 41467_2025_57464_MOESM1_ESM.pdf]

Supporting Information

Lanthanide Single-Atom Catalysts for Efficient CO_2-to-CO Electroreduction

Qiyou Wang[1,2], Tao Luo[1,2], Xueying Cao[3], Yujie Gong[4], Yuxiang Liu[1], Yusen Xiao[1], Hongmei Li[1], Franz Gröbmeyer[5], Ying-Rui Lu[6], Ting-Shan Chan[6], Chao Ma[7], Kang Liu[1], Junwei Fu[1], Shiguo Zhang[7], Changxu Liu[8], Zhang Lin[2], Liyuan Chai[2], Emiliano Cortes[5]*, Min Liu[1]*

[1]Hunan Joint International Research Center for Carbon Dioxide Resource Utilization, State Key Laboratory of Powder Metallurgy, School of Physics, Central South University, Changsha 410083, P. R. China.

[2]School of Metallurgy and Environment, Central South University, Changsha 410083, Hunan, P. R. China.

[3]College of Materials Science and Engineering, Linyi University, Linyi 276000, Shandong, P. R. China.

[4]School of Electrical Engineering, University of South China, Hengyang 421001, Hunan, P. R. China.

[5]Nanoinstitute Munich, Faculty of Physics, Ludwig-Maximilians-Universität (LMU), Munich 80539, Germany.

[6]National Synchrotron Radiation Research Center, 300 Hsinchu, Taiwan.

[7]College of Materials Science and Engineering, Hunan University, Changsha 410082, P. R. China.

[8]Centre for Metamaterial Research & Innovation, Department of Engineering, University of Exeter, Exeter EX4 4QF, UK.

*emails: Emiliano.Cortes@lmu.de, minliu@csu.edu.cn

Table of Contents

DFT calculations S3

Schematic illustration of the preparation S5

XRD patterns S6

High-resolution XPS spectra S7

Raman spectra S8

Nitrogen sorption isotherm and Pore size distribution S9

SEM, HRTEM images and L_3-edge XANES spectra for catalysts S10

Schematic representation of operando ATR-IR spectra S28

Digital photograph of operando XAS equipment S29

operando ATR-IR spectra for Ca SAC S30

CO_2 TPD curves S31

i-t curves for H-cell S32

Spectra of Gas chromatograph for catalysts S33

^{1}HNMR spectra for catalysts S34

Mass spectrometry for catalysts S35

CO_2 electroreduction performance for catalysts in H-cell S36

Schematic representation of electrode on flow-cell reactor S43

CO_2 electroreduction performance for catalysts in flow cell S44

Performance comparison S49

Metal contents of catalysts S50

EXAFS data fitting results of catalysts S51

Detected substances for operando ATR-IR spectra S52

Performance data for catalysts in flow cell and MEA S53

Performance comparison and details S57

References S66

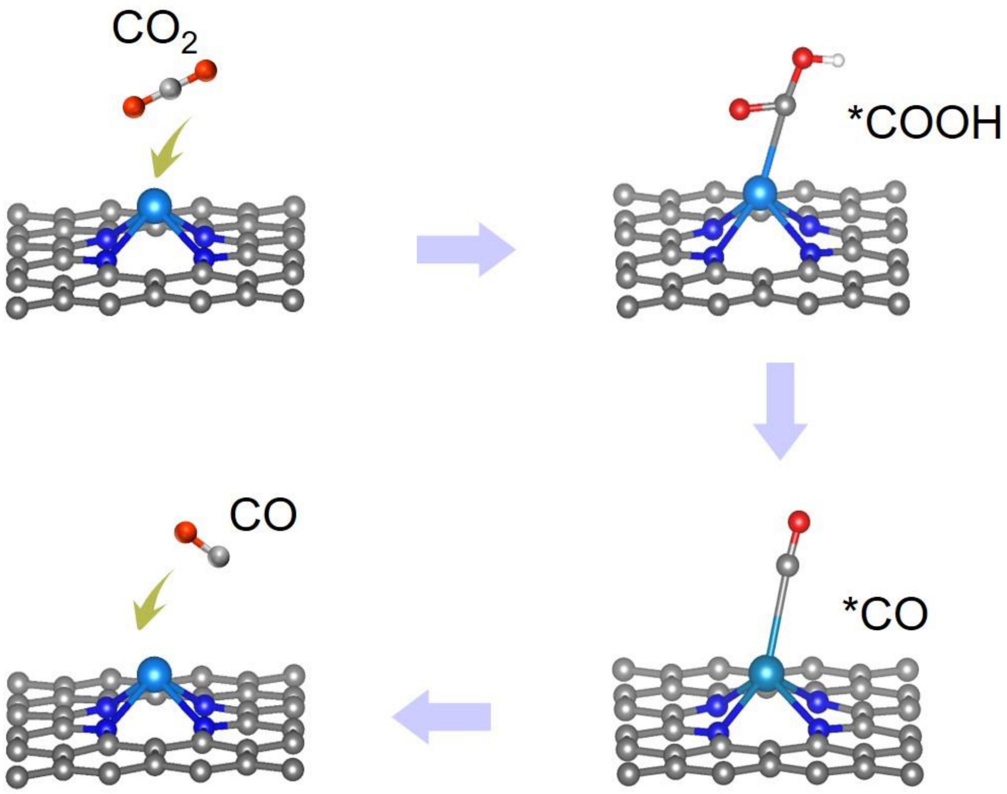

Supplementary Fig. 1 | Structure and adsorption configurations of key intermediates on Ca SAC. Atom color-coding: Red, oxygen; white, hydrogen; grey, carbon; blue, nitrogen; light blue, Ca.

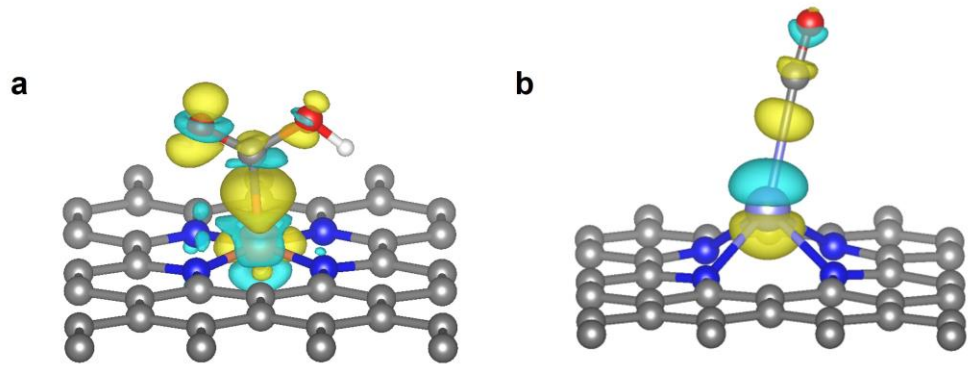

Supplementary Fig. 2 | Bader charge transfer analysis for *COOH absorbed on Fe SAC (**a**) and *CO absorbed on Ca SAC (**b**). Yellow, charge density accumulation; green, charge density depletion. Atom color-coding: Red, oxygen; white, hydrogen; grey, carbon; blue, nitrogen; light blue, Ca; orange, Fe.

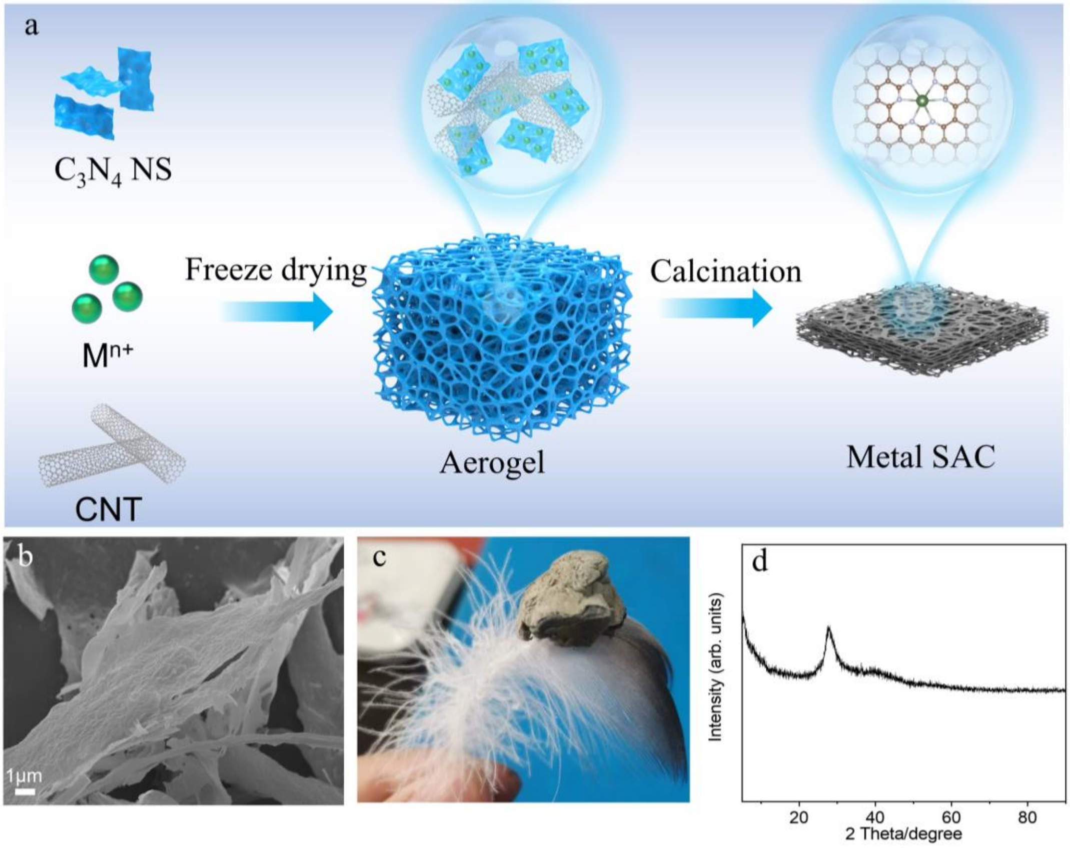

Supplementary Fig. 3 | a Schematic illustration of the preparation for metal SAC. **b** SEM images of C_3N_4 nanosheets. **c** Digital photograph of aerogel. **d** XRD patterns of C_3N_4 nanosheets.

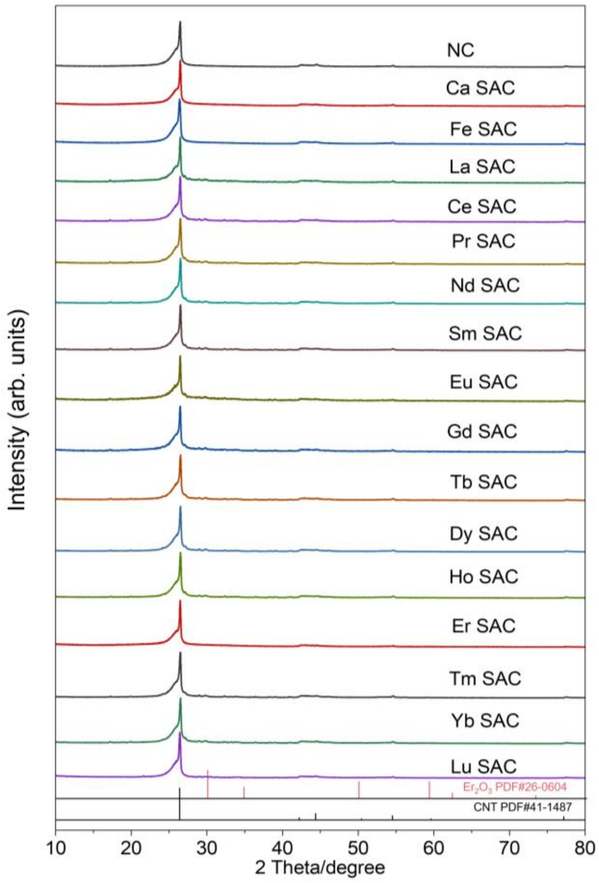

Supplementary Fig. 4 | XRD patterns of catalysts.

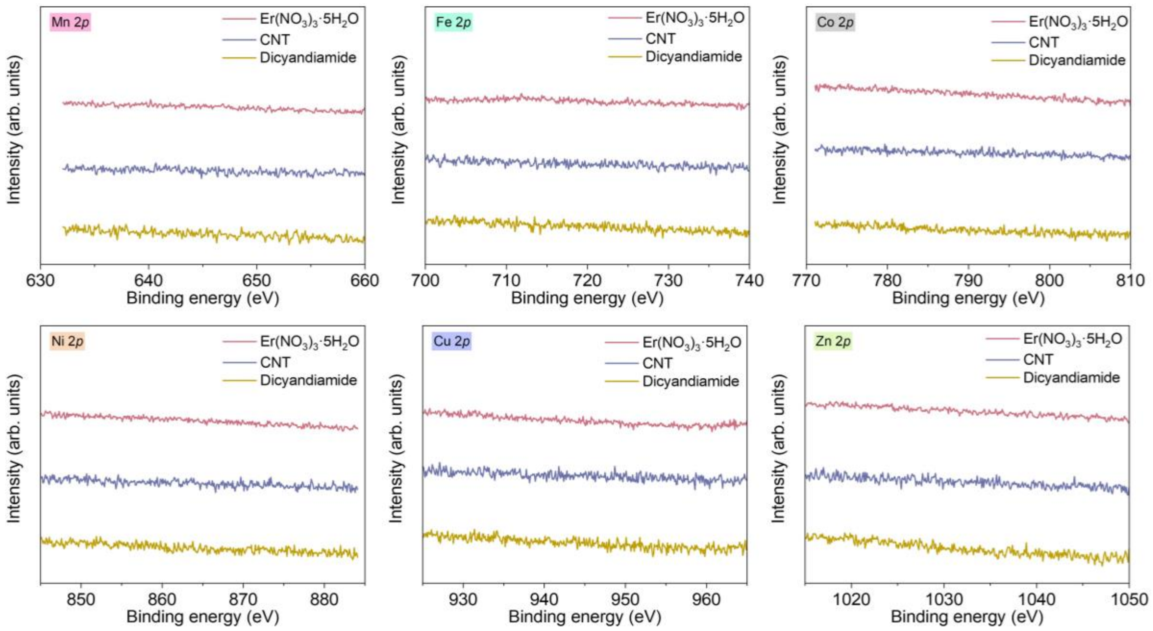

Supplementary Fig. 5 | High-resolution XPS spectra of Mn 2*p*, Fe 2*p*, Co 2*p*, Ni 2*p*, Cu 2*p*, and Zn 2*p* for the raw materials.

As shown in Supplementary Fig. 5, no XPS signal of Mn 2*p*, Fe 2*p*, Co 2*p*, Ni 2*p*, Cu 2*p*, and Zn 2*p* is detected in all the precursors, indicating that the metal contamination is neglected.

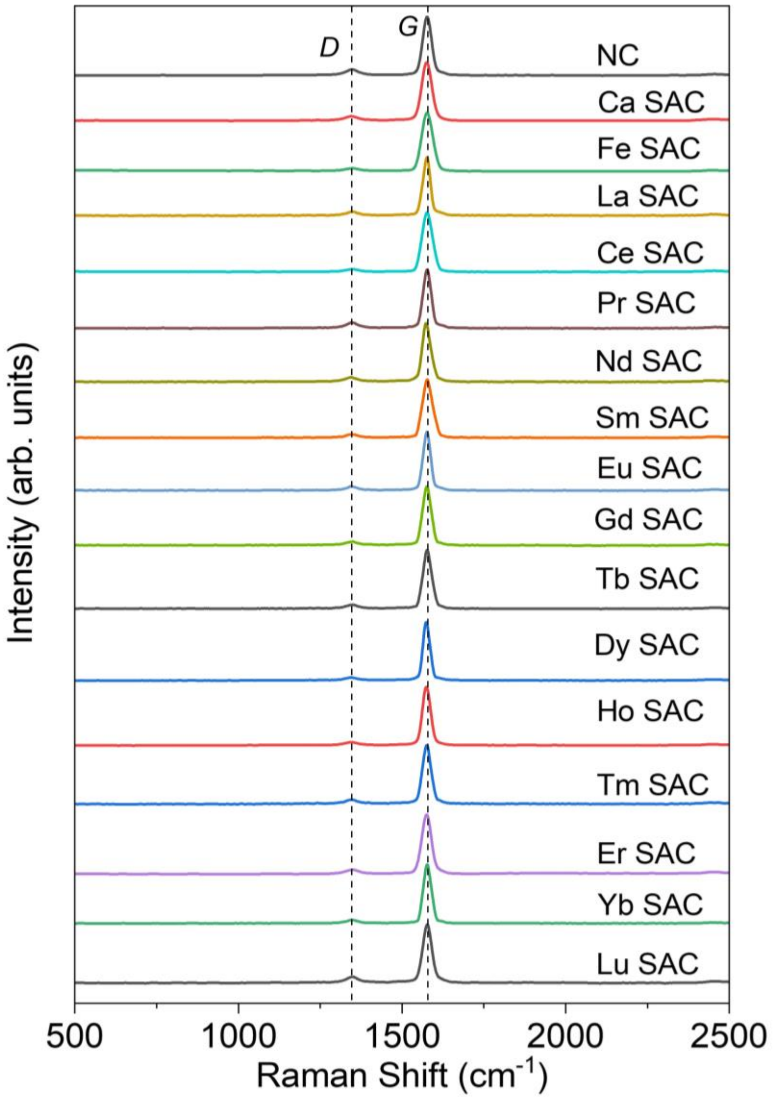

Supplementary Fig. 6 | Raman spectra of catalysts. The peaks at 1344 and 1574 cm^{-1} are attributed to the *D* band and *G* band of CNT, respectively.

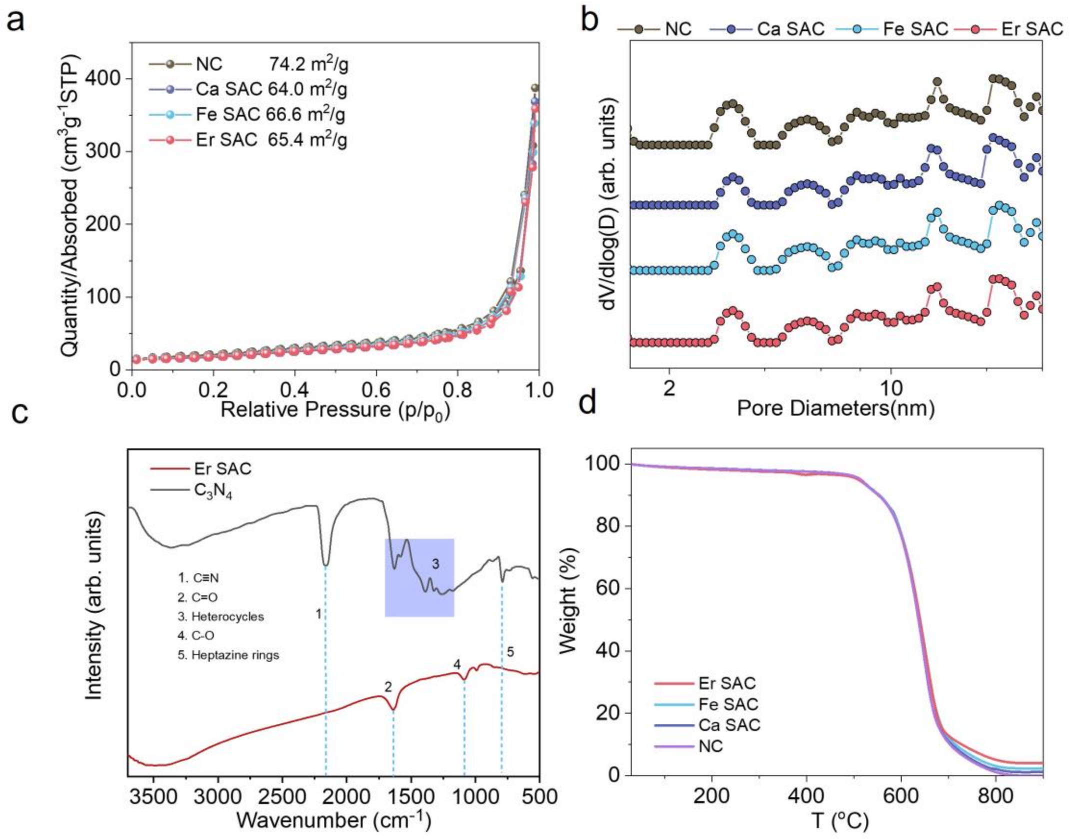

Supplementary Fig. 7 | a Nitrogen sorption isotherm for different catalysts. **b** Pore size distribution of different catalysts. **c** FT-IR spectra of C_3N_4 NS and Er SAC. **d** TGA curves of different catalysts in air.

NC, Er SAC, Fe SAC and Ca SAC show high similarity in specific surface and pore size distribution. The characteristic peak of C_3N_4 disappeared after pyrolysis.

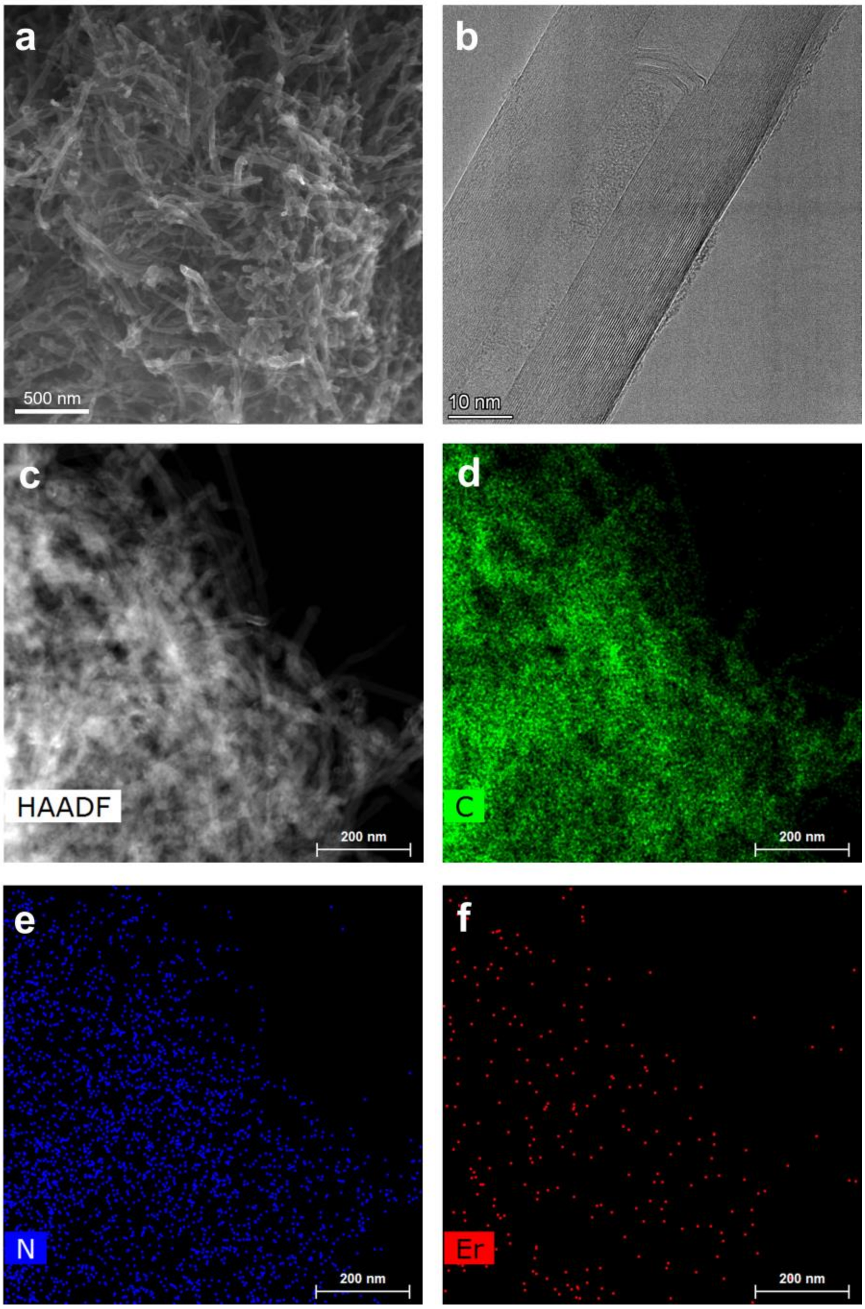

Supplementary Fig. 8 | Structural characterization of Er SAC. a SEM image. **b** HRTEM image. **c-f** EDS mapping image.

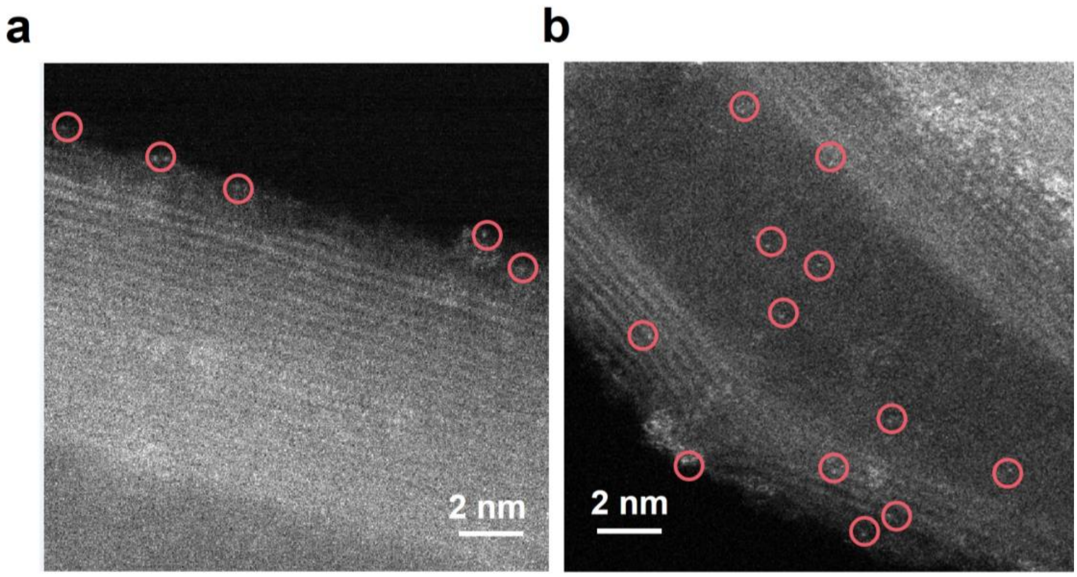

Supplementary Fig. 9 | AC HAADF-STEM images of Ca SAC (**a**) and Fe SAC (**b**).

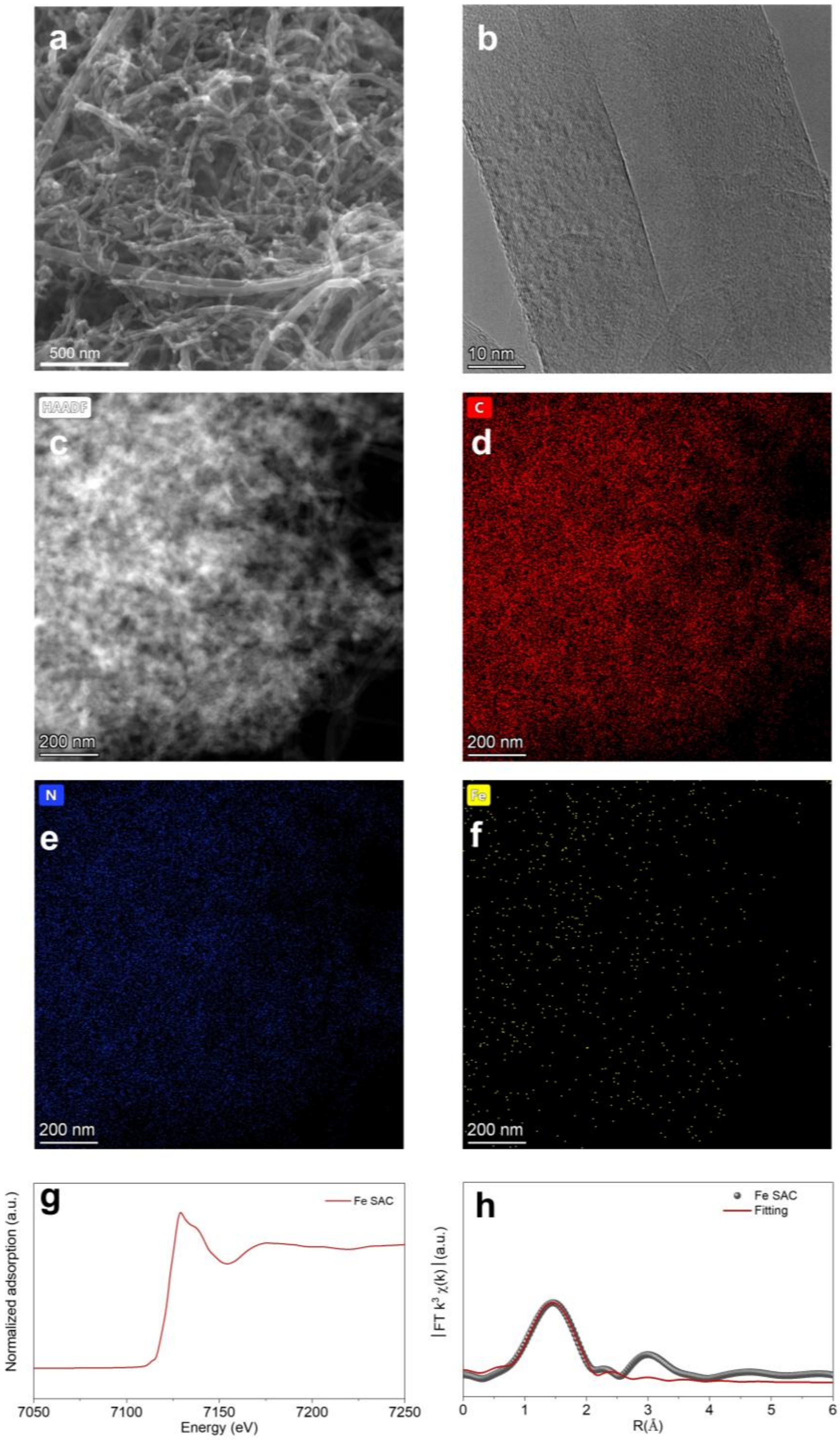

Supplementary Fig. 10 | Structural characterization of Fe SAC. a SEM image. **b** HRTEM image. **c-f** EDS mapping image. **g** K-edge XANES spectra. **h** the corresponding EXAFS R space fitting curve.

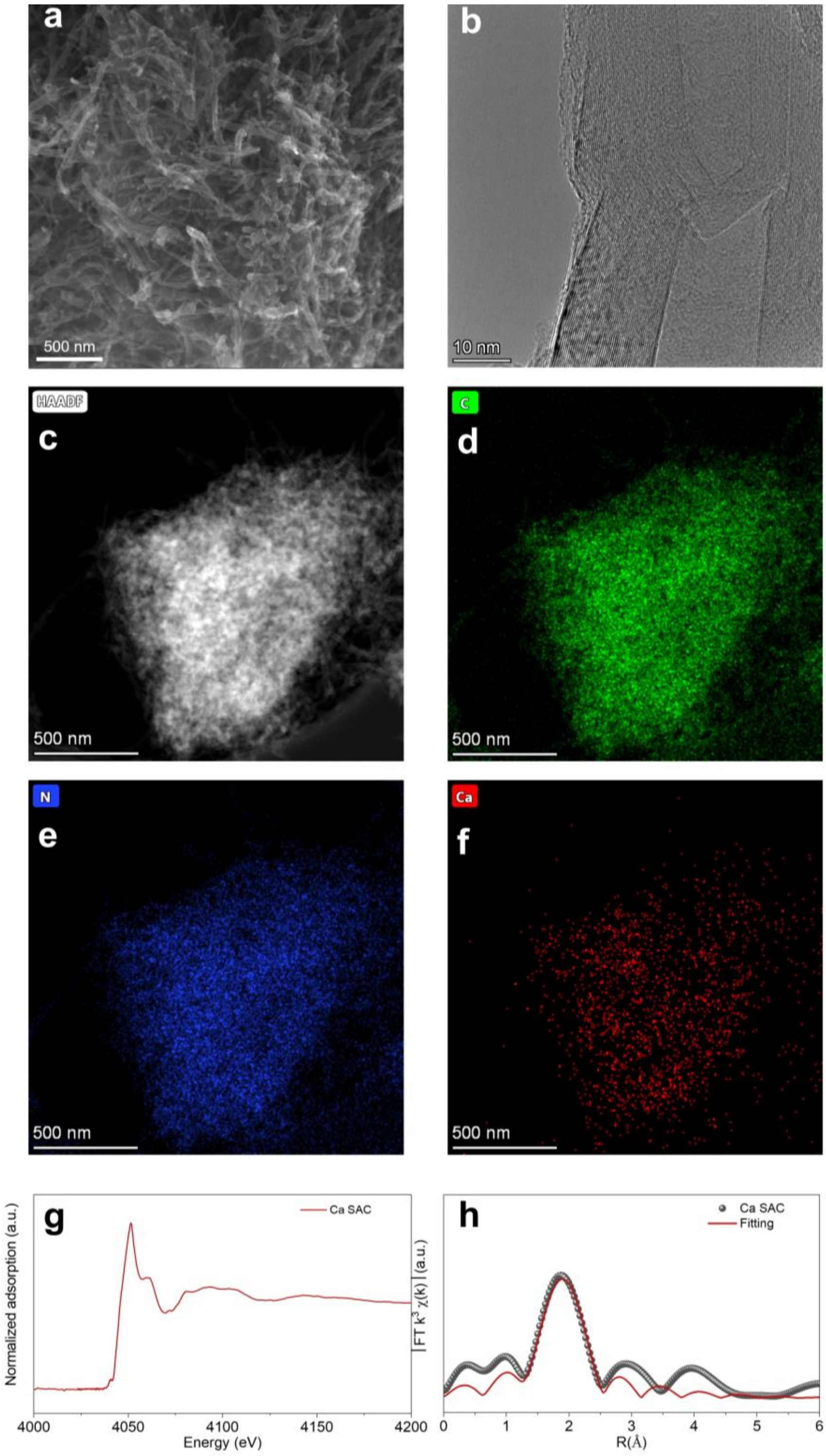

Supplementary Fig. 11 | Structural characterization of Ca SAC. a SEM image. **b** HRTEM image. **c-f** EDS mapping image. **g** K-edge XANES spectra. **h** the corresponding EXAFS R space fitting curve.

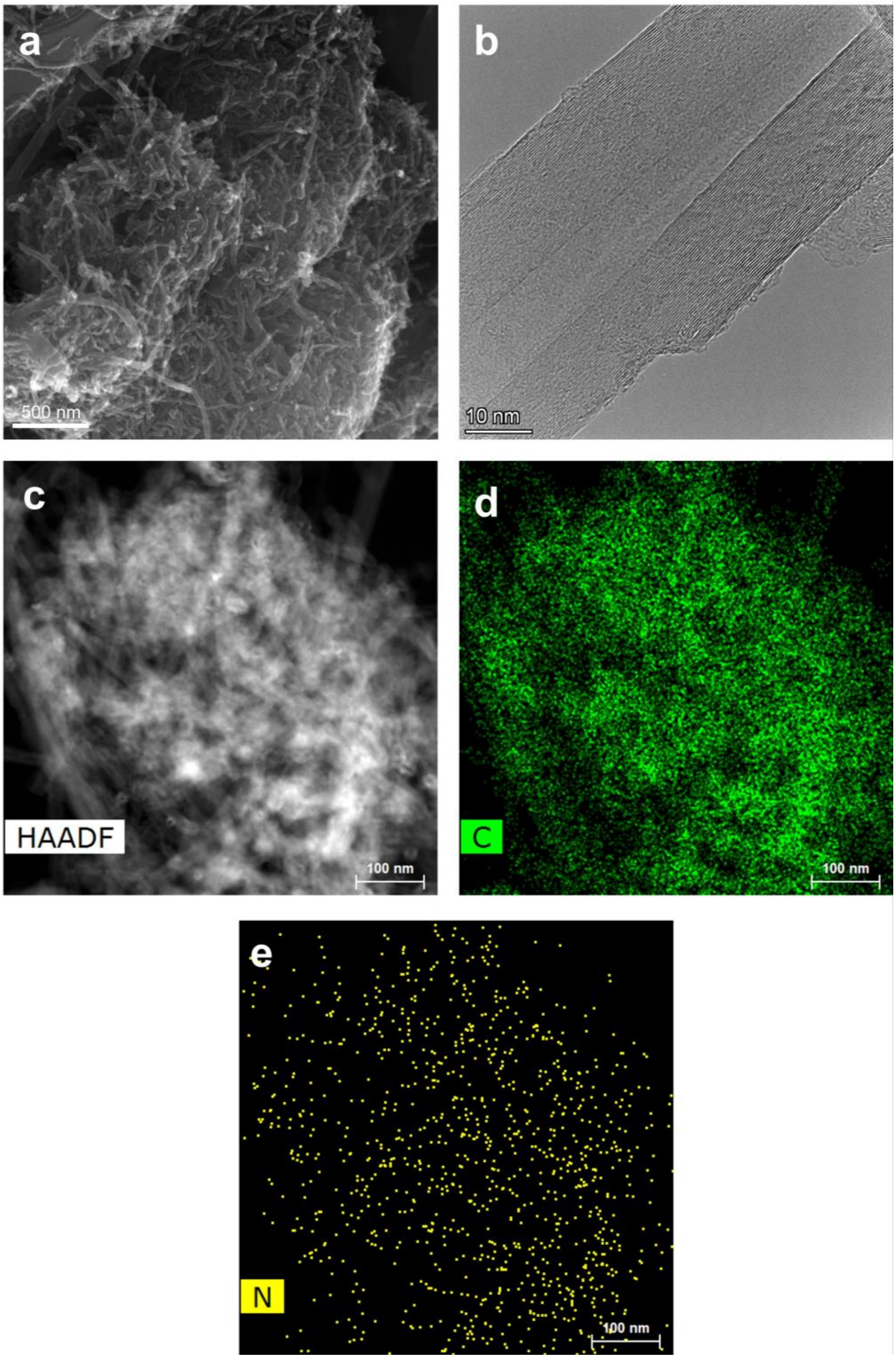

Supplementary Fig. 12 | Structural characterization NC. a SEM image. **b** HRTEM image. **c-e** EDS mapping image.

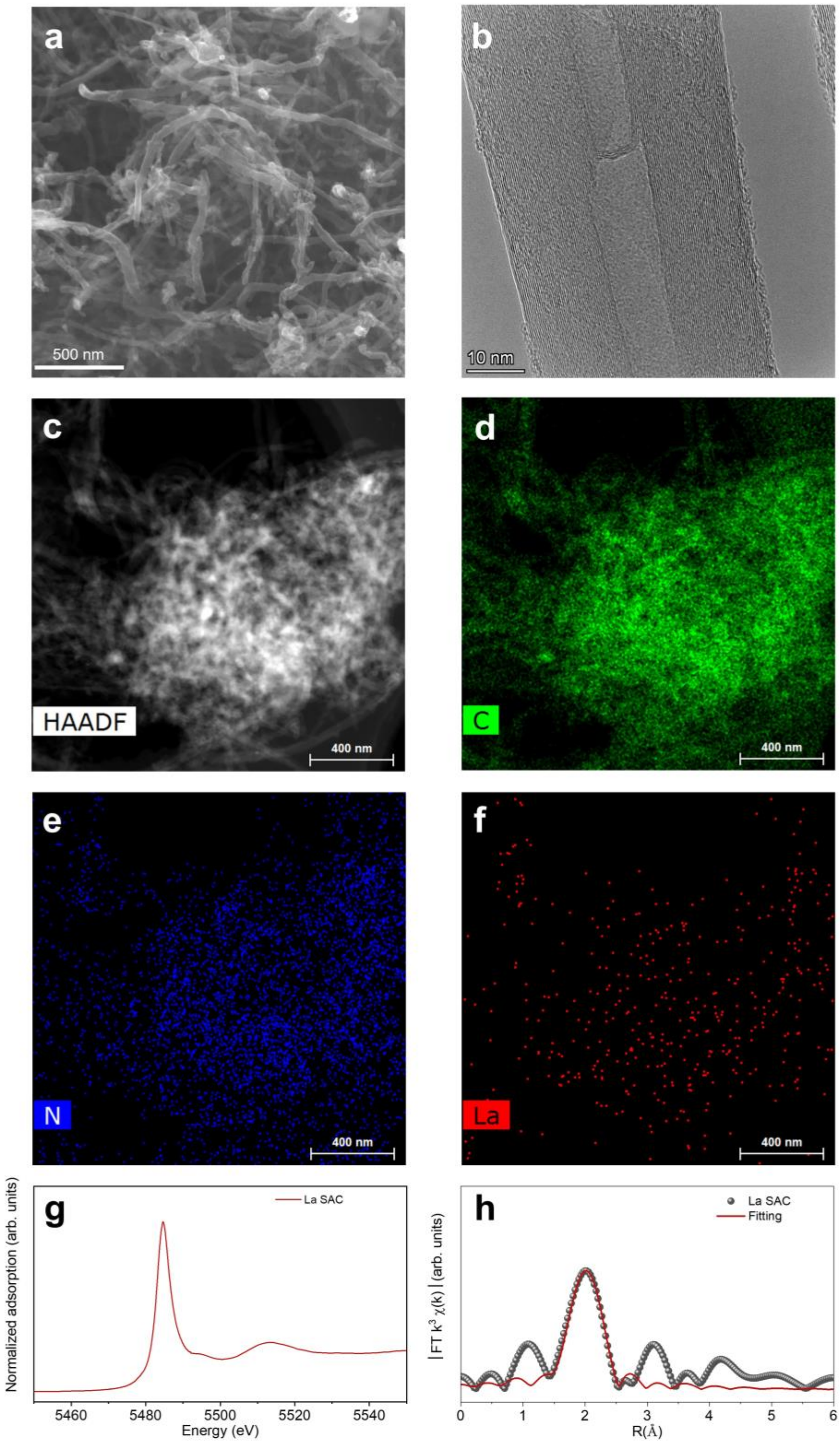

Supplementary Fig. 13 | Structural characterization of La SAC. a SEM image. **b** HRTEM image. **c-f** EDS mapping image. **g** L_3-edge XANES spectra. **h** the corresponding EXAFS R space fitting curve.

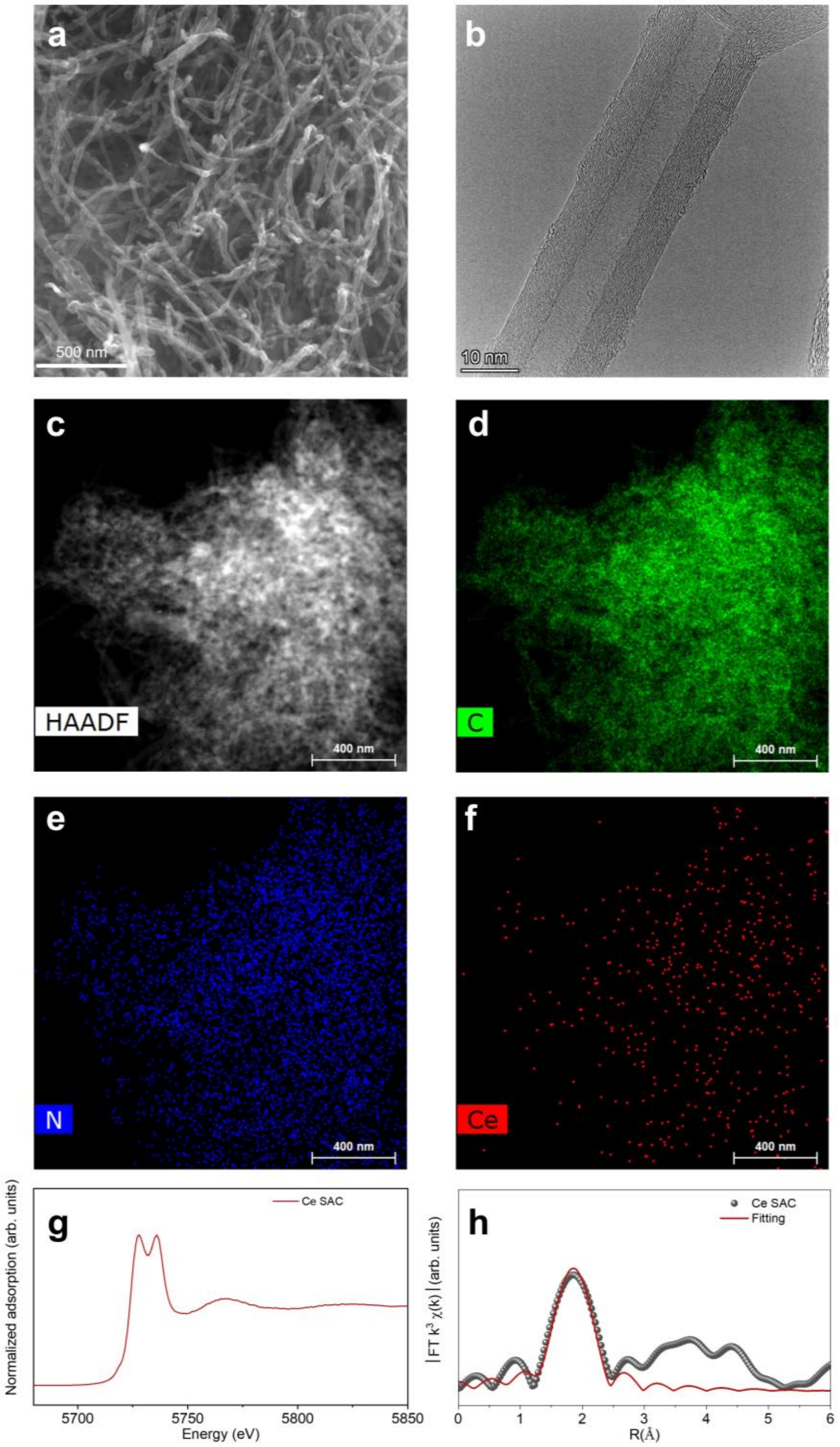

Supplementary Fig. 14 | Structural characterization of Ce SAC. a SEM image. **b** HRTEM image. **c-f** EDS mapping image. **g** L_3-edge XANES spectra. **h** the corresponding EXAFS R space fitting curve.

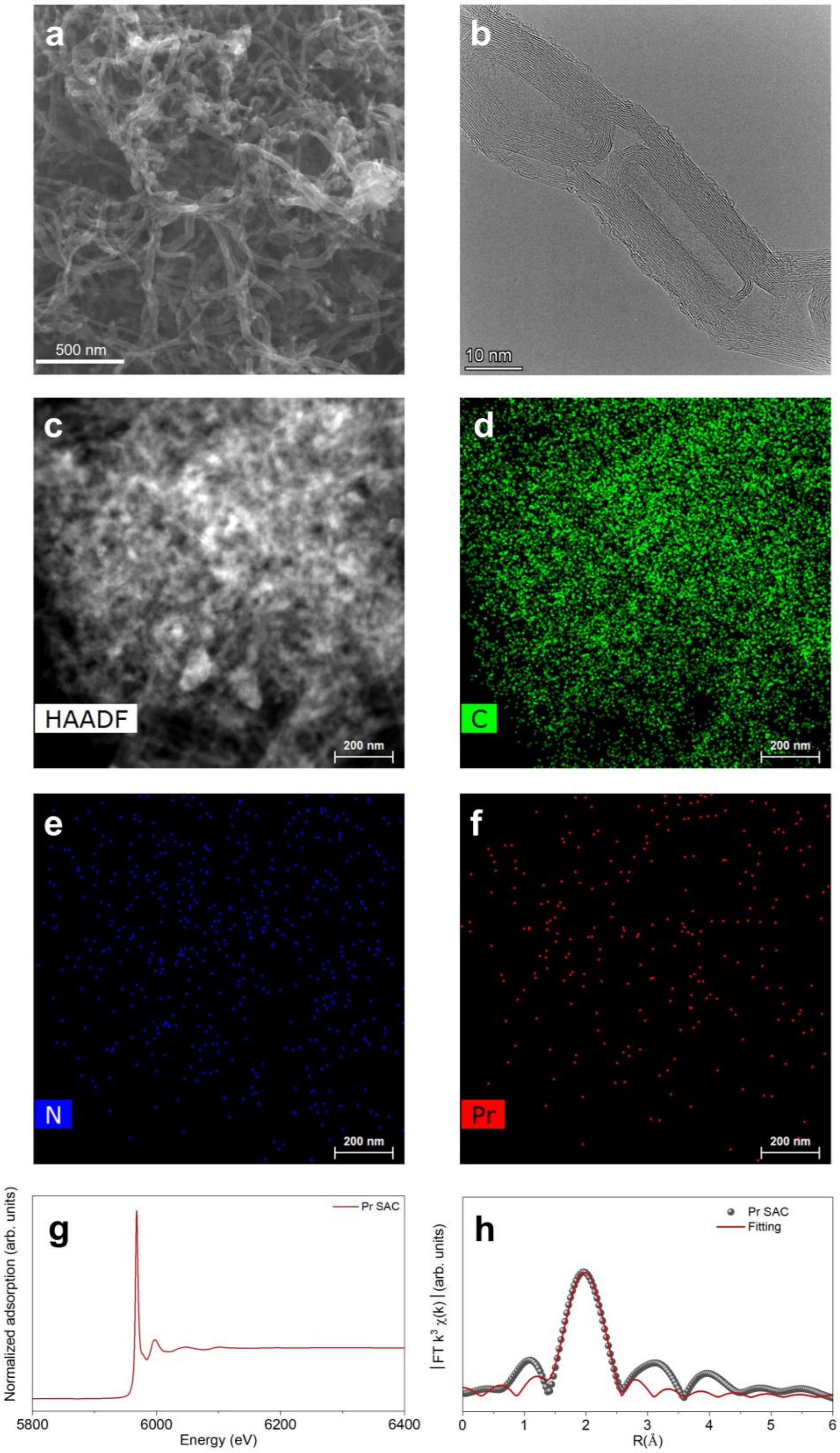

Supplementary Fig. 15 | Structural characterization of Pr SAC. a SEM image. **b** HRTEM image. **c-f** EDS mapping image. **g** L_3-edge XANES spectra. **h** the corresponding EXAFS R space fitting curve.

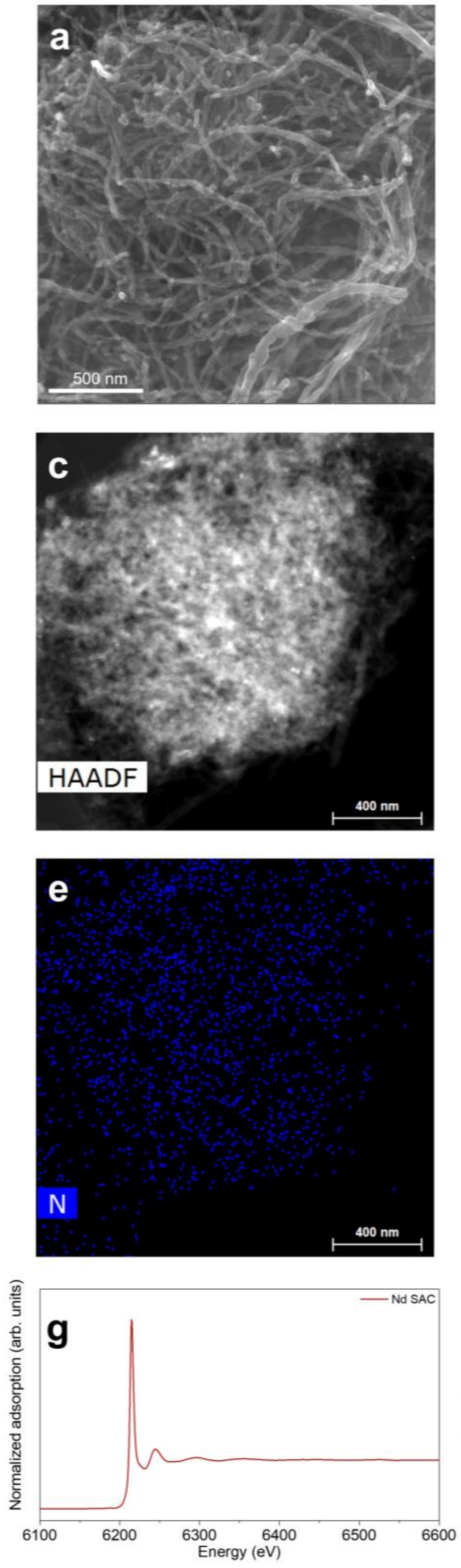

Supplementary Fig. 16 | Structural characterization of Nd SAC. a SEM image. **b** HRTEM image. **c-f** EDS mapping image. **g** L_3-edge XANES spectra. **h** the corresponding EXAFS R space fitting curve.

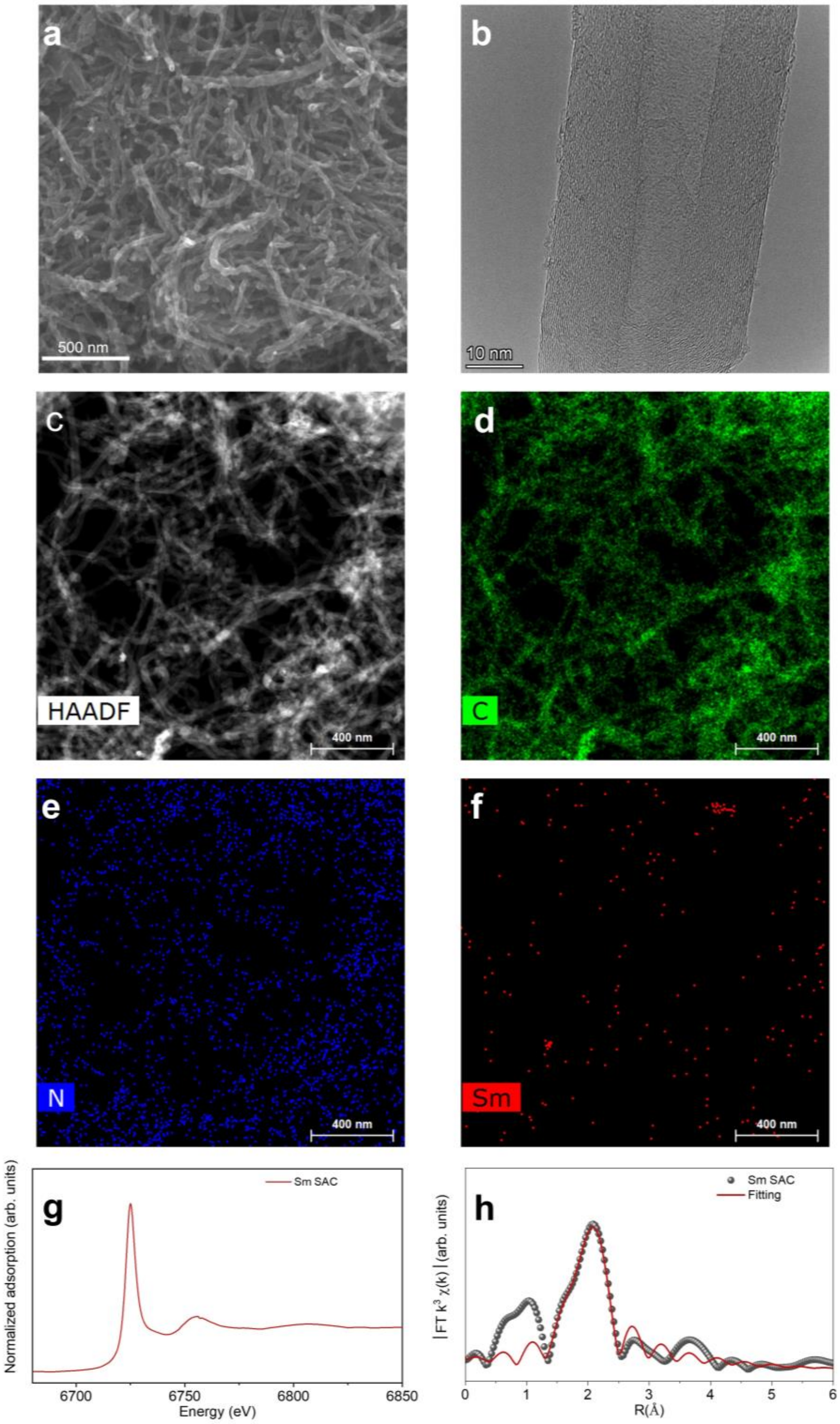

Supplementary Fig. 17 | Structural characterization of Sm SAC. a SEM image. **b** HRTEM image. **c-f** EDS mapping image. **g** L_3-edge XANES spectra. **h** the corresponding EXAFS R space fitting curve.

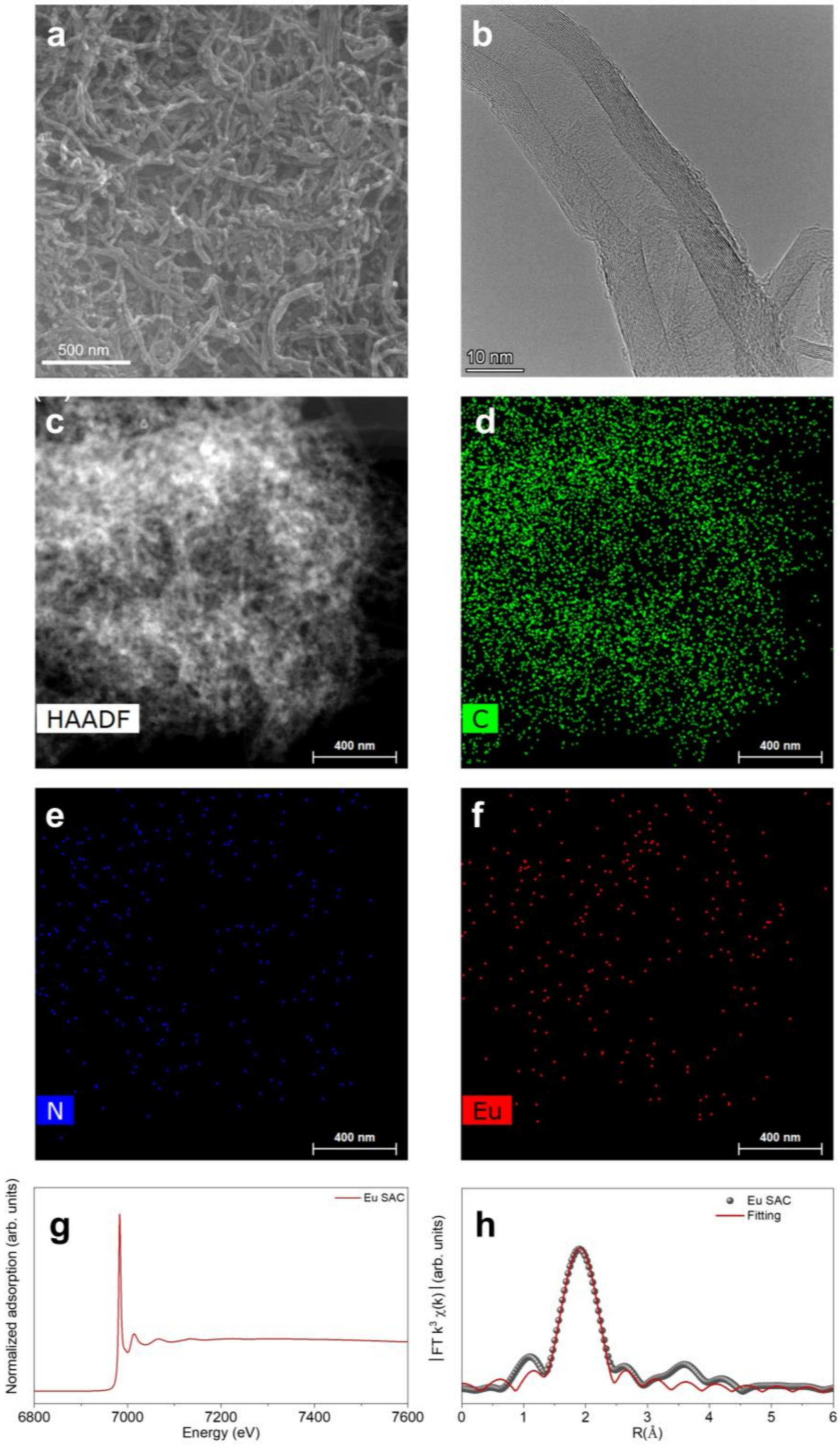

Supplementary Fig. 18 | Structural characterization of Eu SAC. a SEM image. **b** HRTEM image. **c-f** EDS mapping image. **g** L_3-edge XANES spectra. **h** the corresponding EXAFS R space fitting curve.

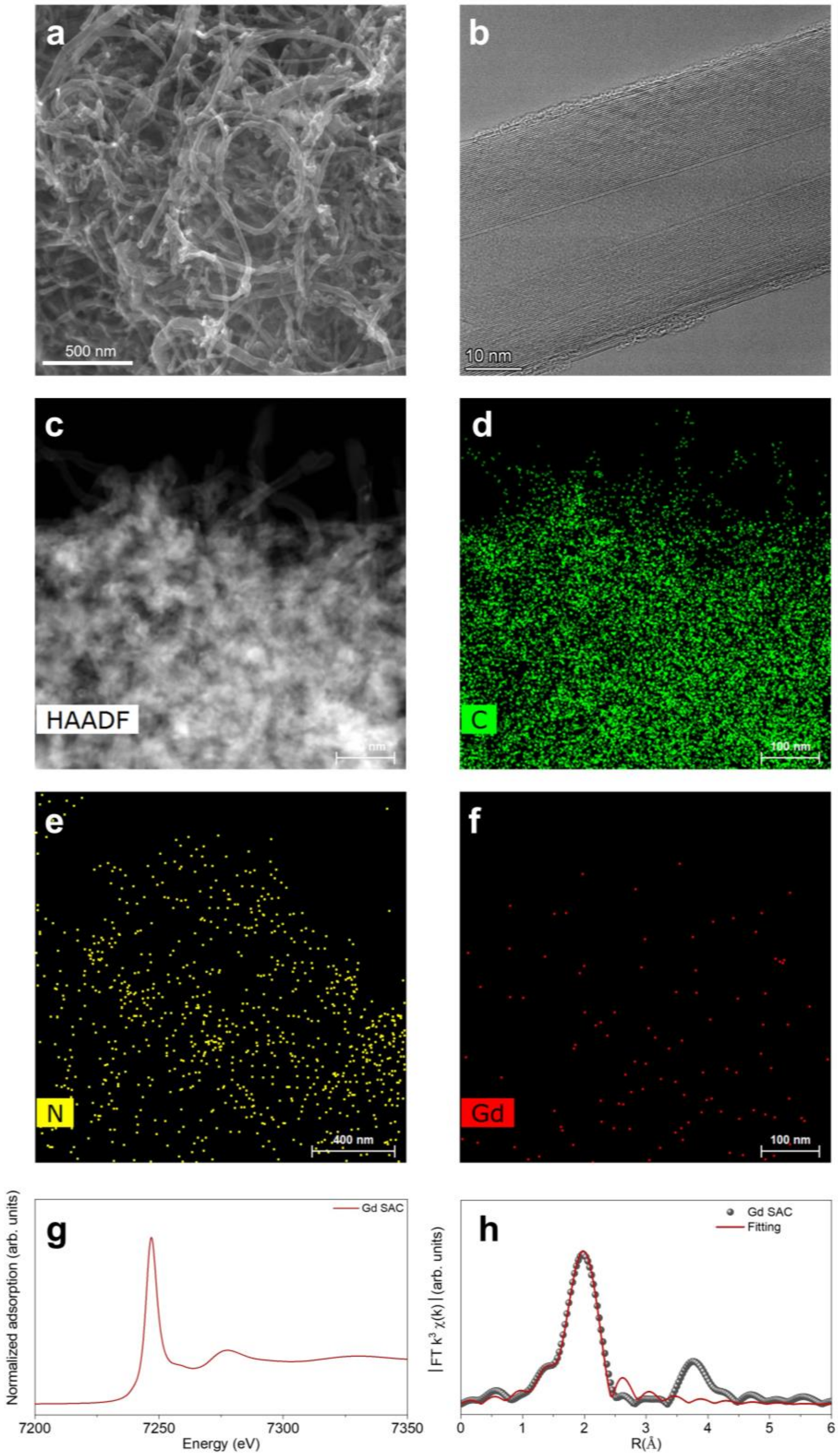

Supplementary Fig. 19 | Structural characterization of Gd SAC. a SEM image. **b** HRTEM image. **c-f** EDS mapping image. **g** L_3-edge XANES spectra. **h** the corresponding EXAFS R space fitting curve.

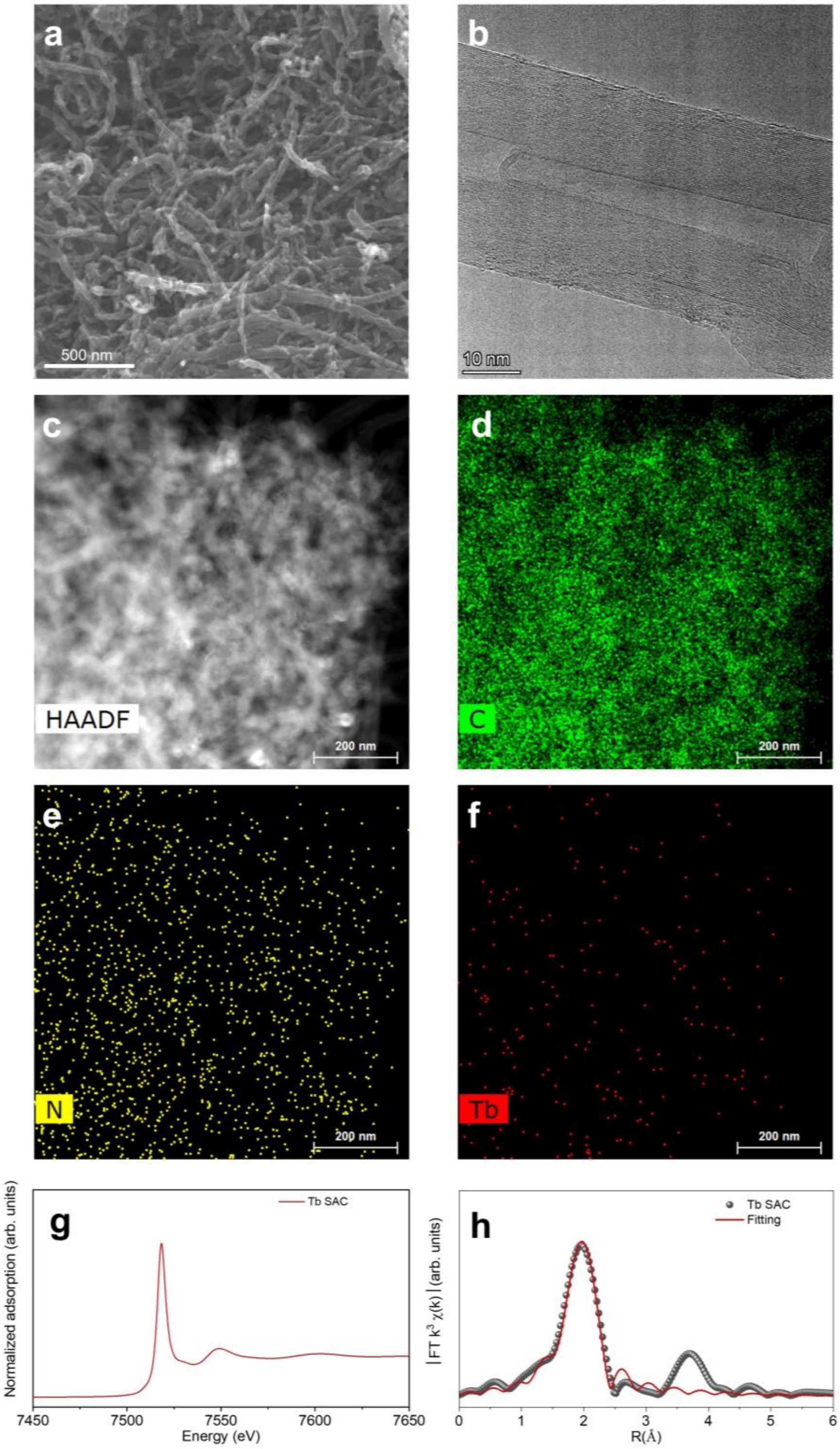

Supplementary Fig. 20 | Structural characterization of Tb SAC. a SEM image. **b** HRTEM image. **c-f** EDS mapping image. **g** L_3-edge XANES spectra. **h** the corresponding EXAFS R space fitting curve.

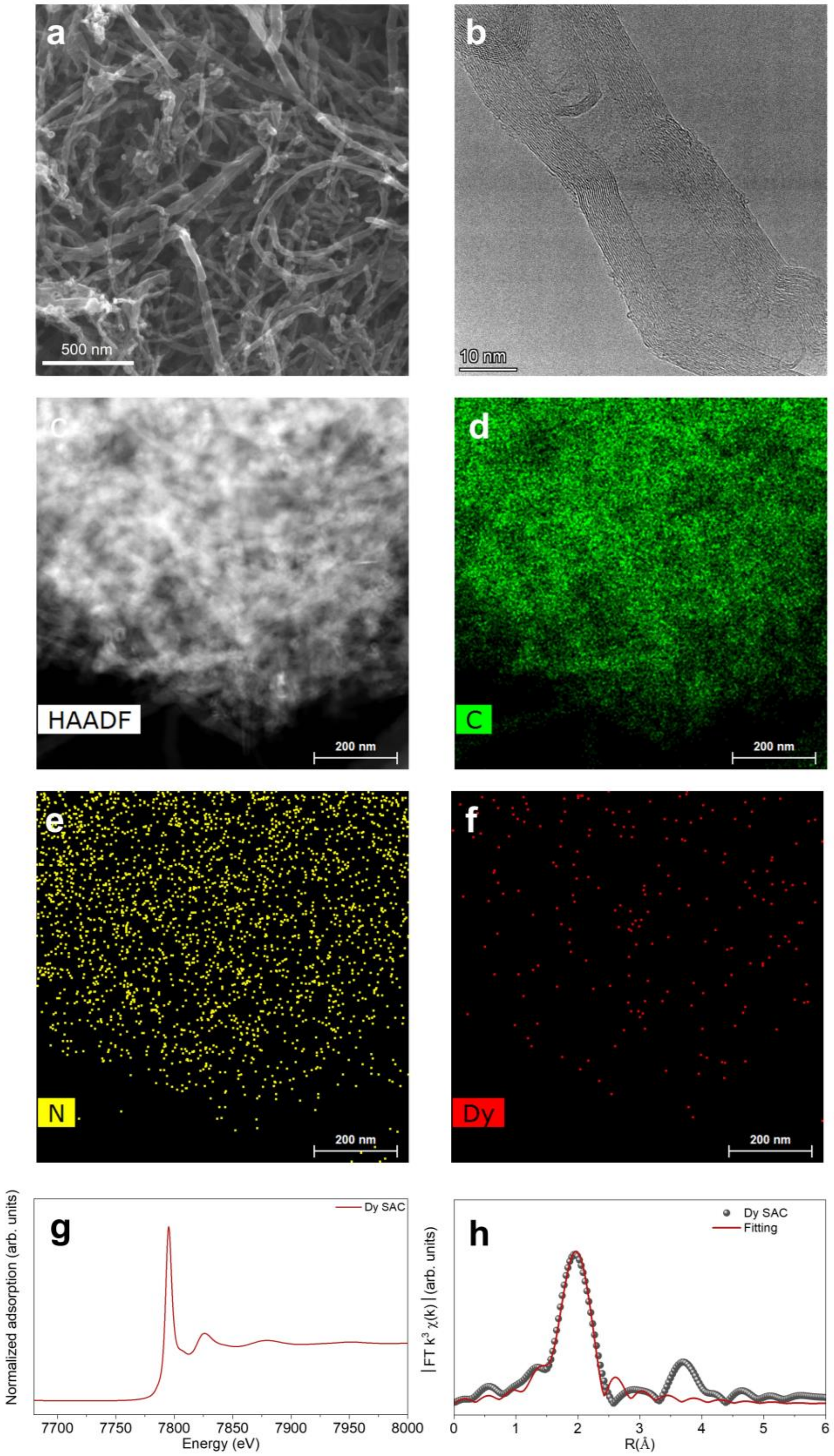

Supplementary Fig. 21 | Structural characterization of Dy SAC. a SEM image. **b** HRTEM image. **c-f** EDS mapping image. **g** L_3-edge XANES spectra. **h** the corresponding EXAFS R space fitting curve.

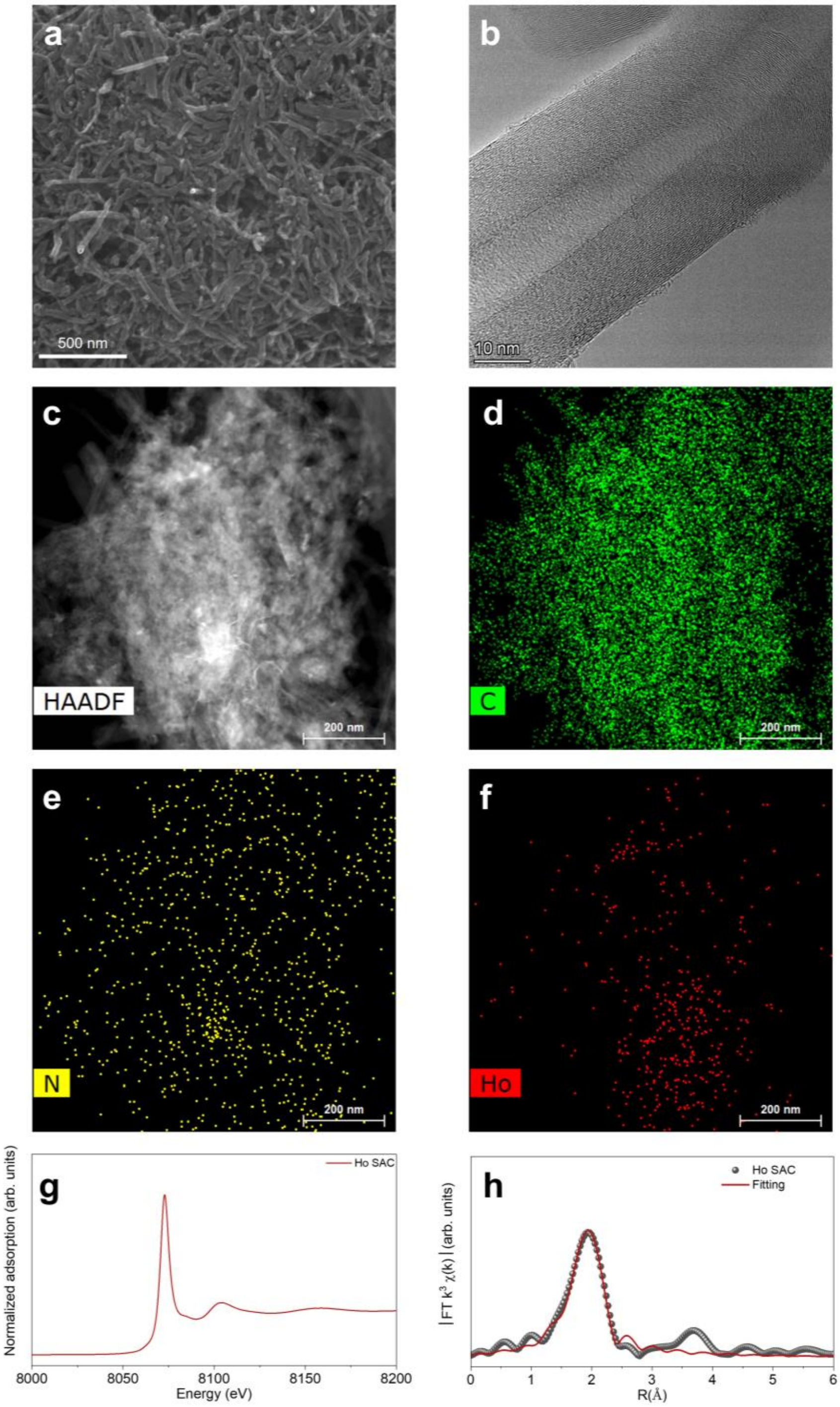

Supplementary Fig. 22 | Structural characterization of Ho SAC. a SEM image. **b** HRTEM image. **c-f** EDS mapping image. **g** L_3-edge XANES spectra. **h** the corresponding EXAFS R space fitting curve.

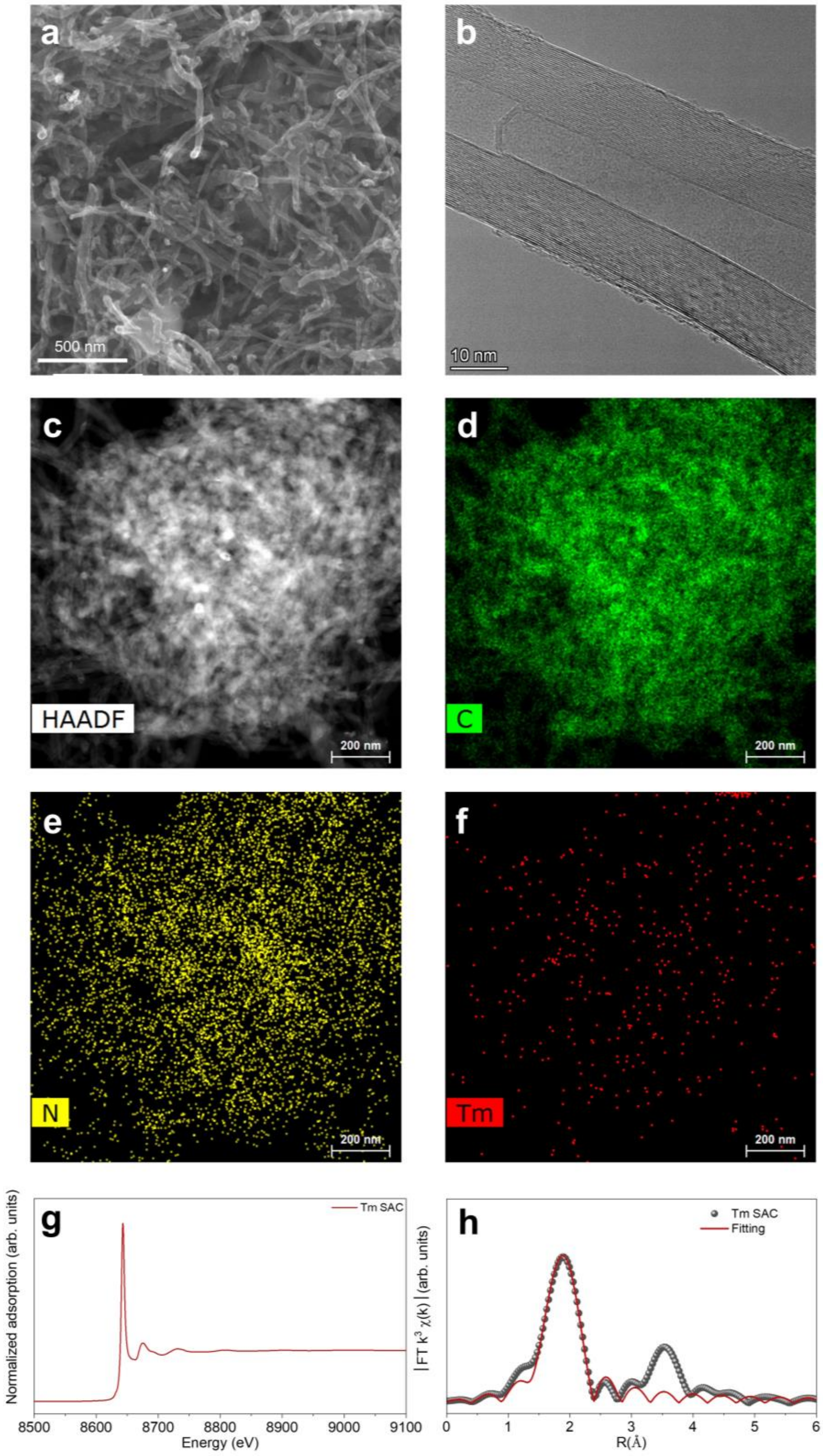

Supplementary Fig. 23 | Structural characterization of Tm SAC. a SEM image. **b** HRTEM image. **c** EDS mapping image. **g** L_3-edge XANES spectra. **h** the corresponding EXAFS R space fitting curve.

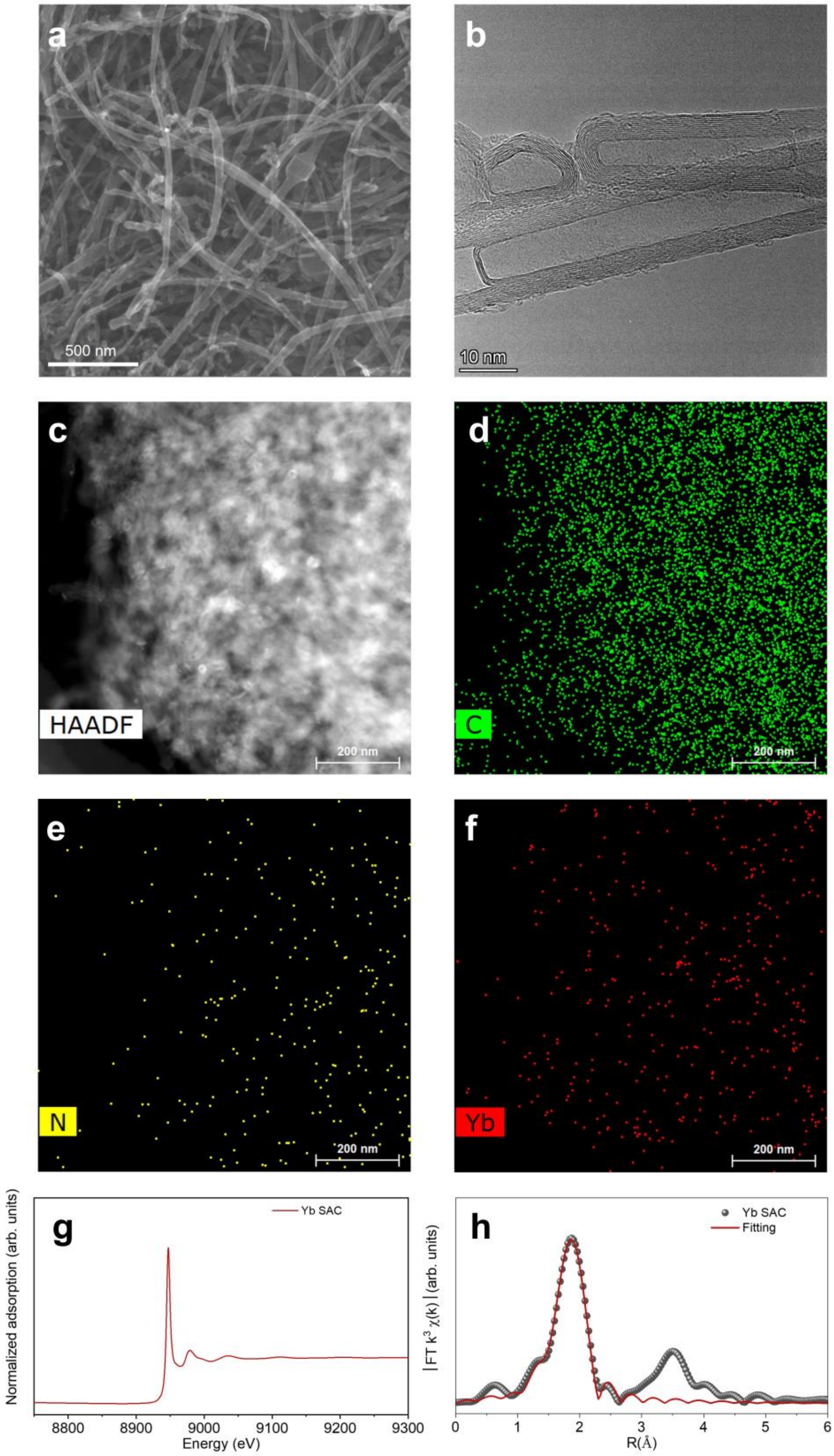

Supplementary Fig. 24 | Structural characterization of Yb SAC. a SEM image. **b** HRTEM image. **c-f** EDS mapping image. **g** L_3-edge XANES spectra. **h** the corresponding EXAFS R space fitting curve.

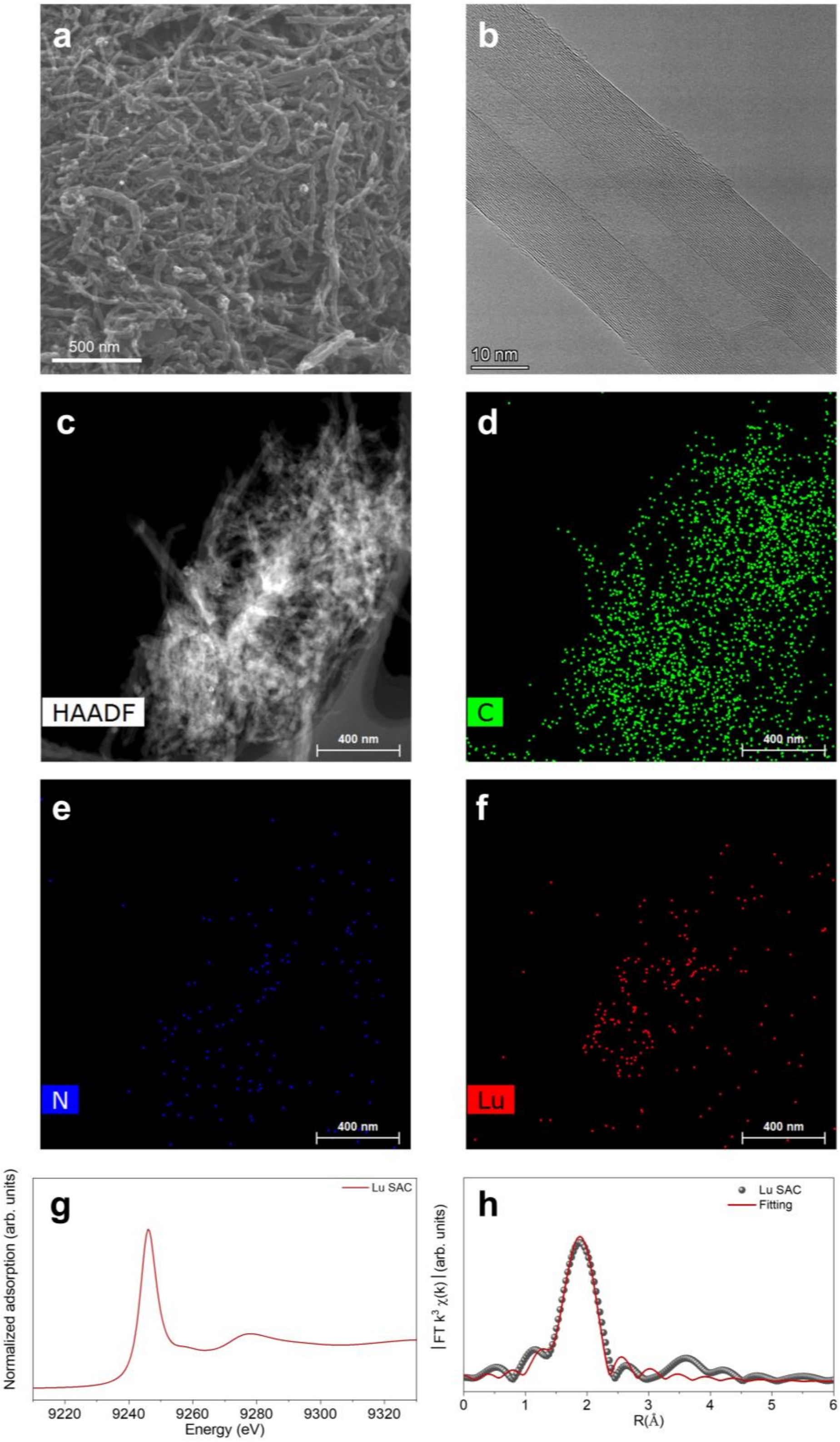

Supplementary Fig. 25 | Structural characterization of Lu SAC. a SEM image. **b** HRTEM image. **c** EDS mapping image. **g** L_3-edge XANES spectrum. **h** the corresponding EXAFS R space fitting curve.

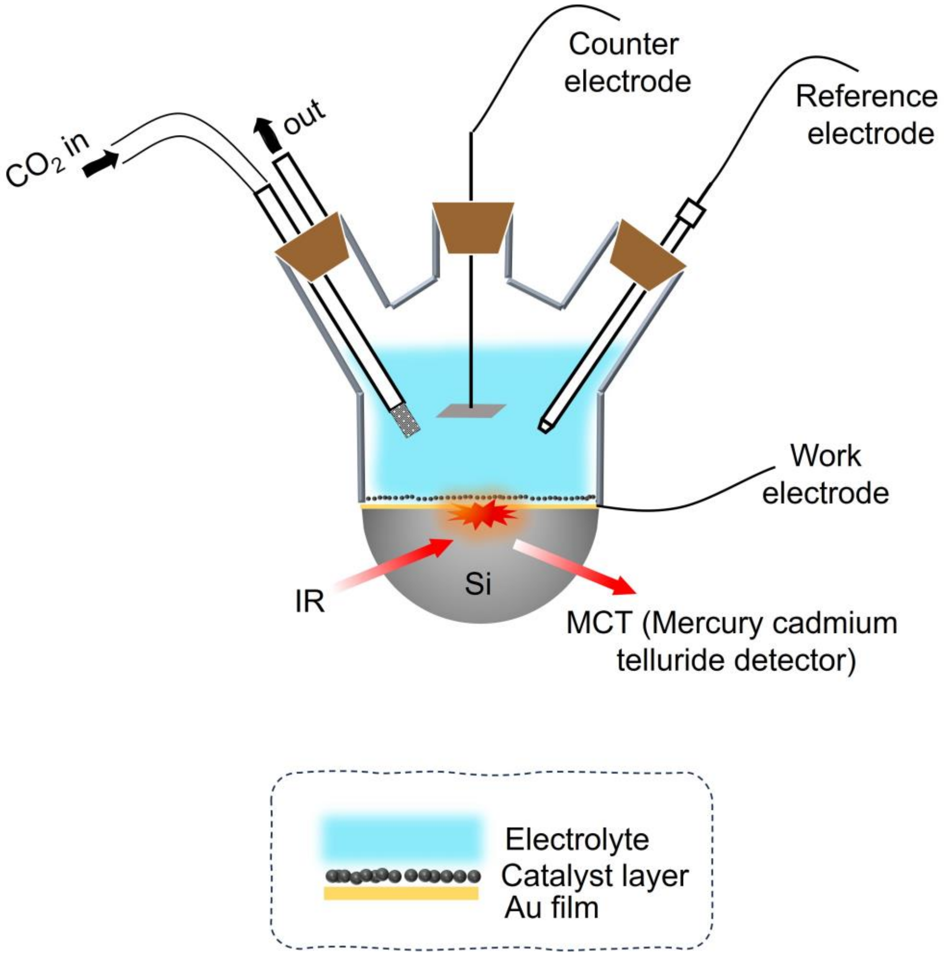

Supplementary Fig. 26 | Schematic representation of operando attenuated total reflection surface-enhanced infrared absorption spectroscopy.

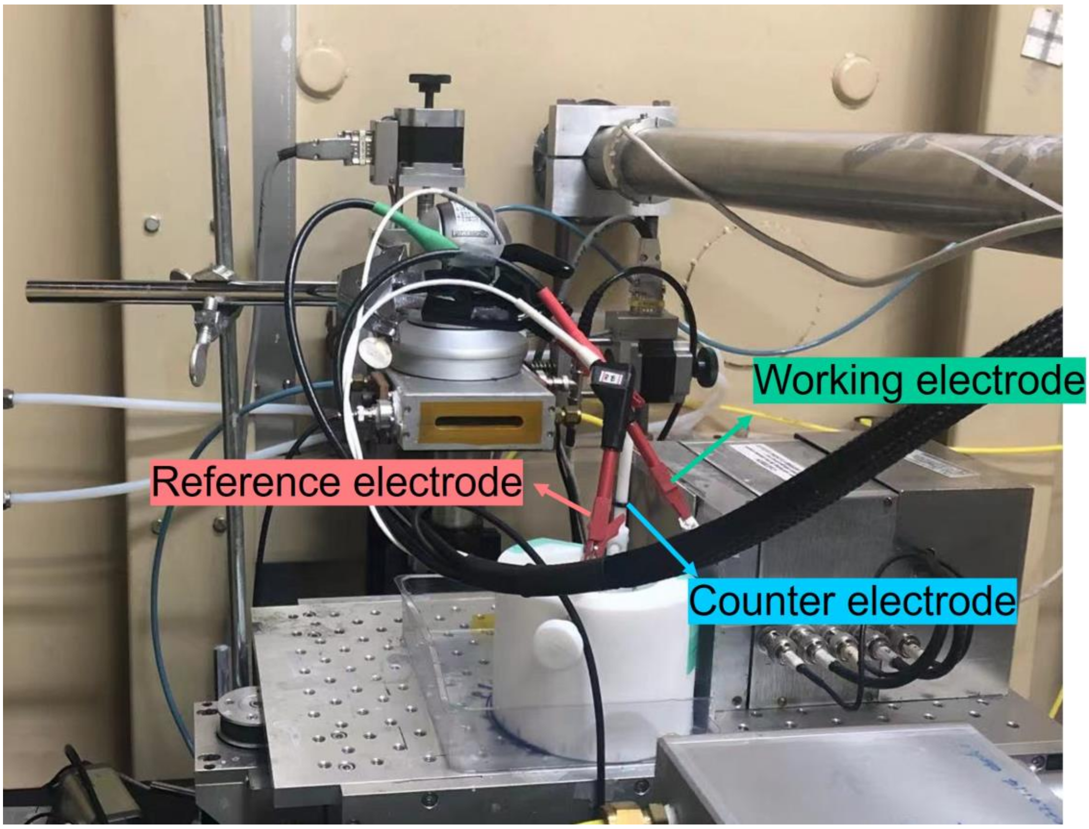

Supplementary Fig. 27 | Digital photograph of operando XAS equipment.

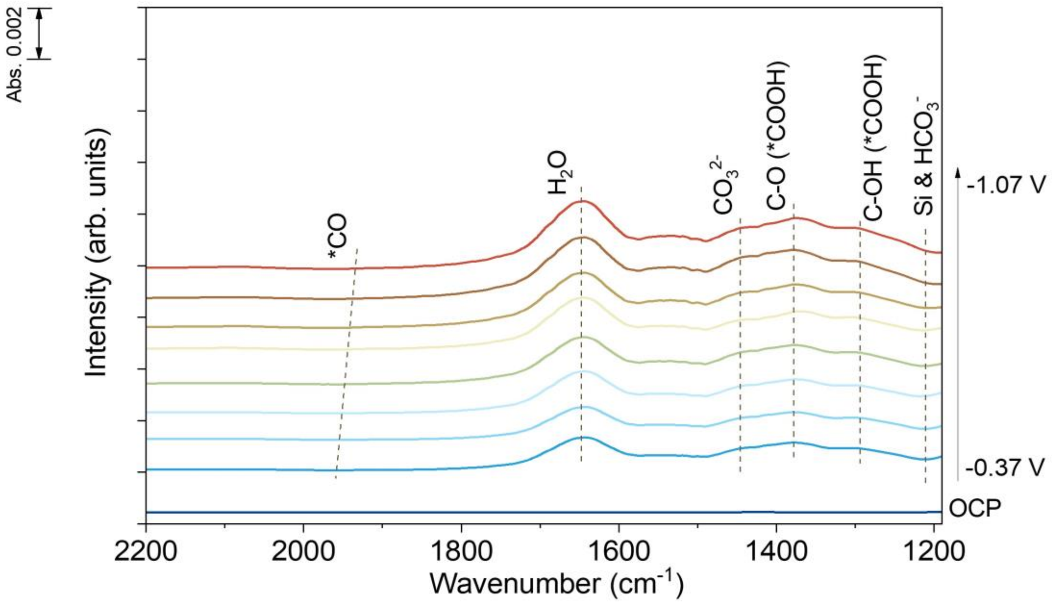

Supplementary Fig. 28 | operando ATR-IR spectra of Ca SAC (without iR-correction). No obvious *CO on Ca SAC was found even though CO was extensively produced, indicating produced CO was well desorbed from Ca sites, consistent with the results from DFT calculations.

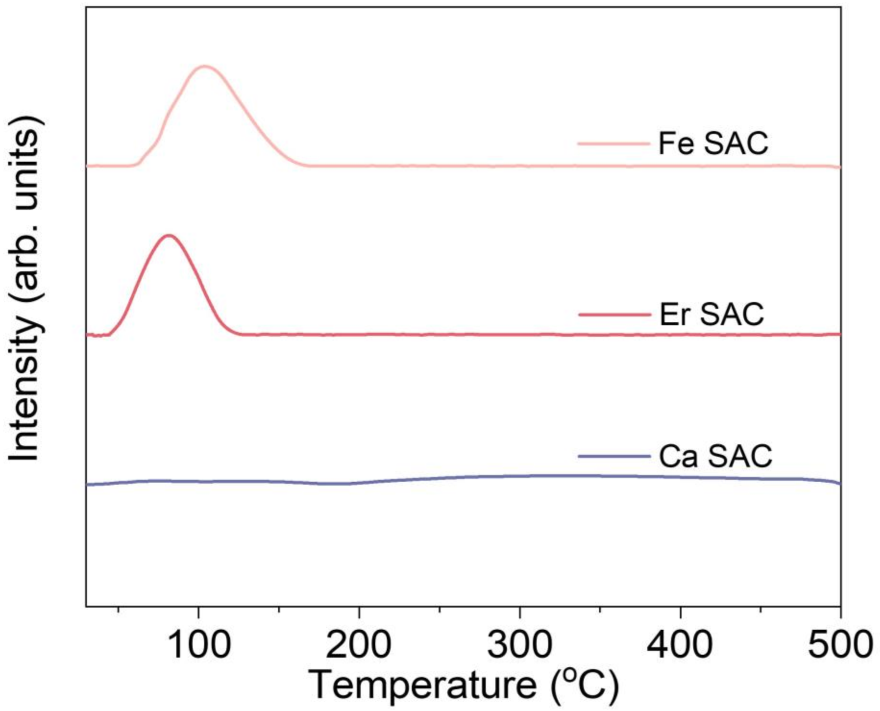

Supplementary Fig. 29 | CO_2 temperature program desorption curves of catalysts.

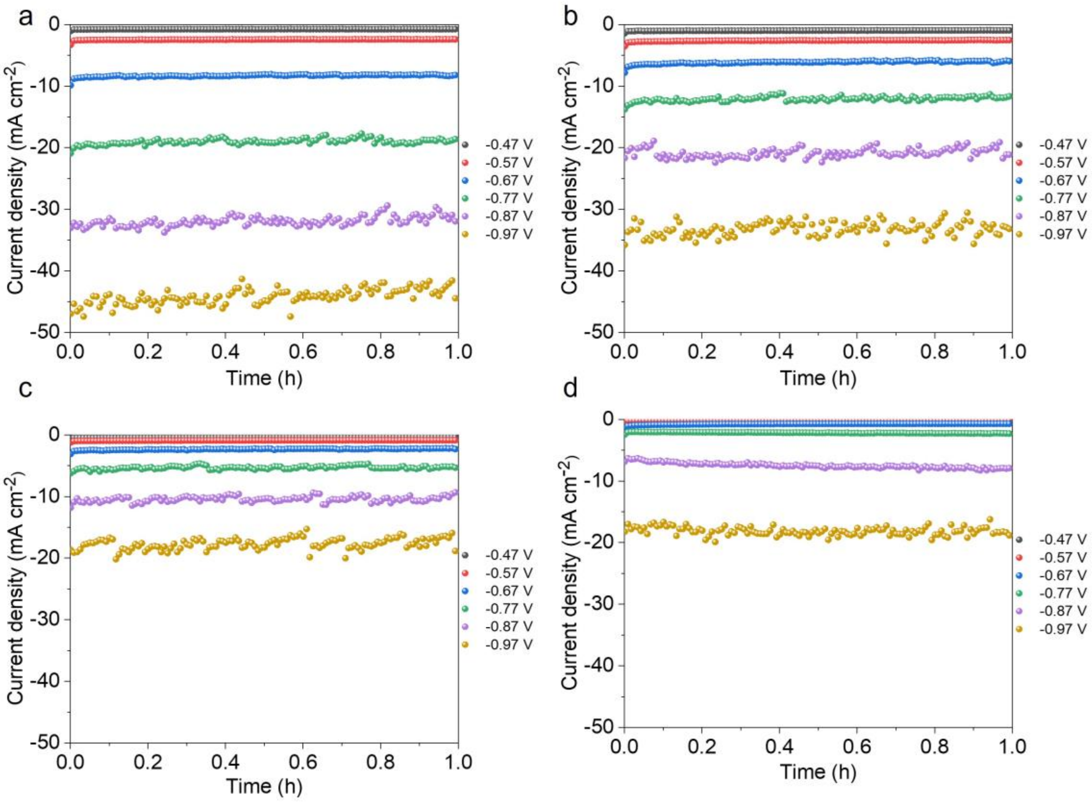

Supplementary Fig. 30 | i-t curves from -0.47 V to −0.97 V vs. RHE in H-cell with CO_2-saturated 0.5 M $KHCO_3$ solution (potentials without iR-correction). **a** Er SAC. **b** Ca SAC. **c** Fe SAC. **d** NC catalyst.

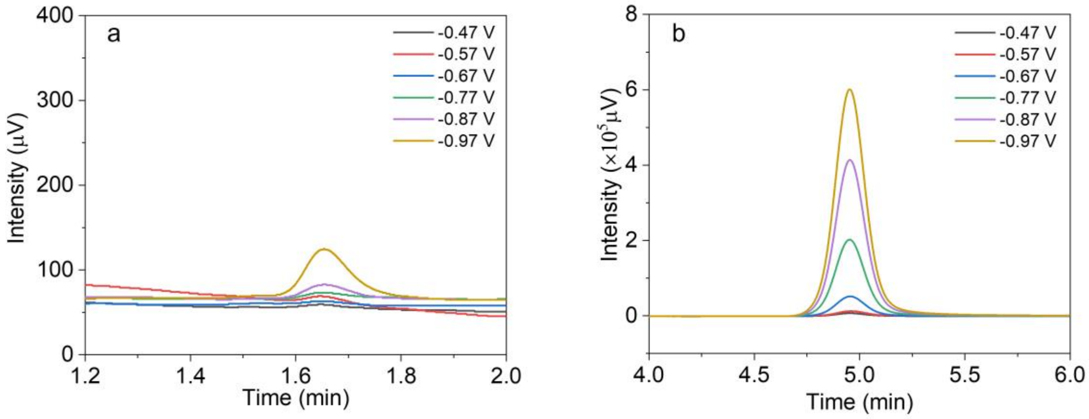

Supplementary Fig. 31 | The spectra of Gas Chromatograph (GC) for **a** H_2 and **b** CO produced by Er SAC in H-Cell (potentials without iR-correction).

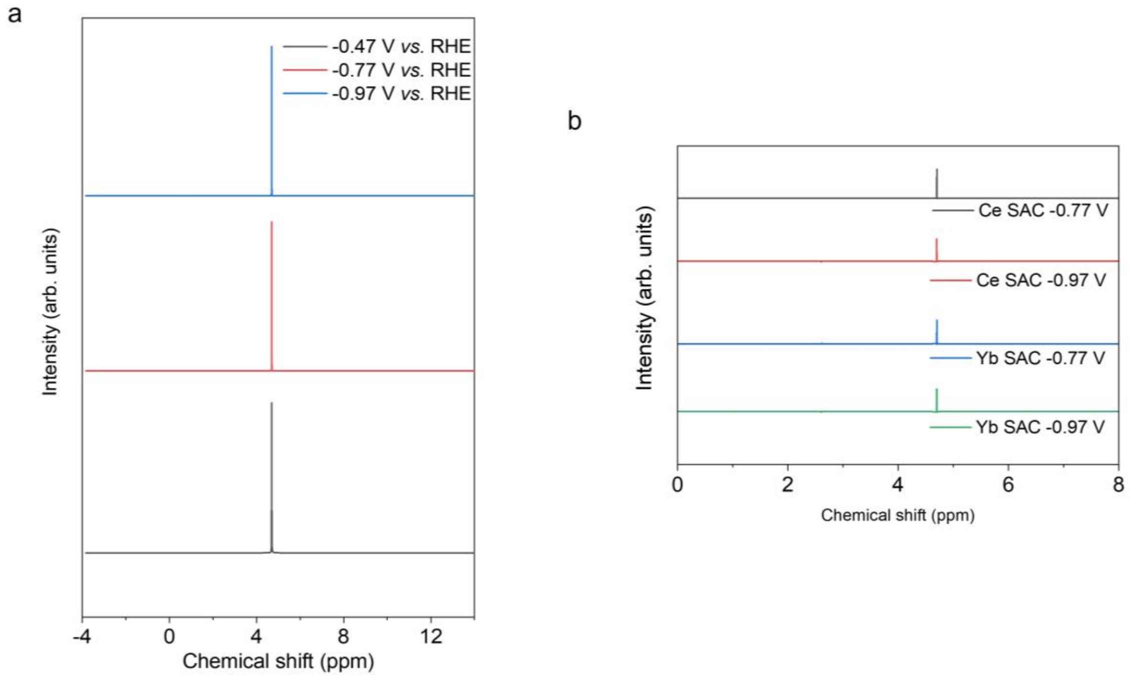

Supplementary Fig. 32 | ^{1}HNMR spectra after CO_2 reduction electrolysis at different potential (without iR-correction) in CO_2 saturated 0.5 M $KHCO_3$ solution for Er SAC (**a**), Ce and Yb SAC (**b**). No C_2 and liquid products (such as ethanol, methanol) was observed by ^{1}H NMR.

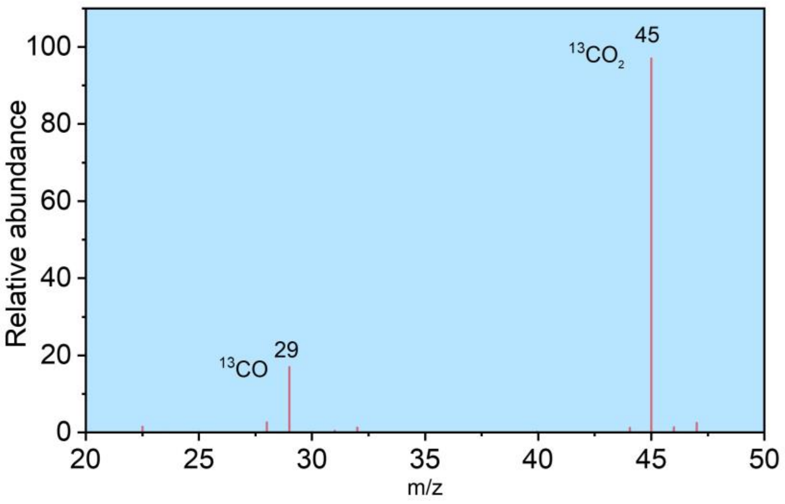

Supplementary Fig. 33 | Mass spectrometry signal of CO_2RR using $^{13}CO_2$ as the feedstock.

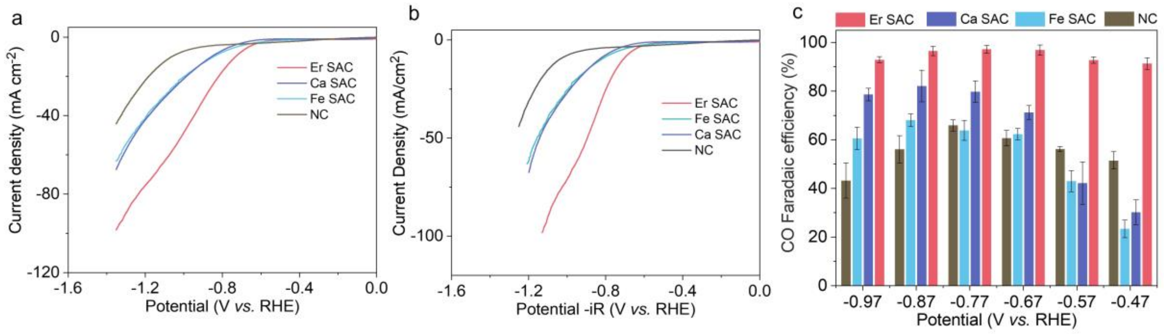

Supplementary Fig. 34 | a LSV curves at scan rate of 5 mV s^{-1} in H-cell with pure CO_2 saturated 0.5 M $KHCO_3$ solution (potentials without iR-correction). **b** LSV curves (potentials with 95% iR-correction, Resistance = 25 ± 0.5 Ω). **c** FE_{CO} at different potentials without IR compensation in H-cell under pure CO_2. The error bars correspond to the standard deviations of measurements over three separately prepared samples under the same testing conditions.

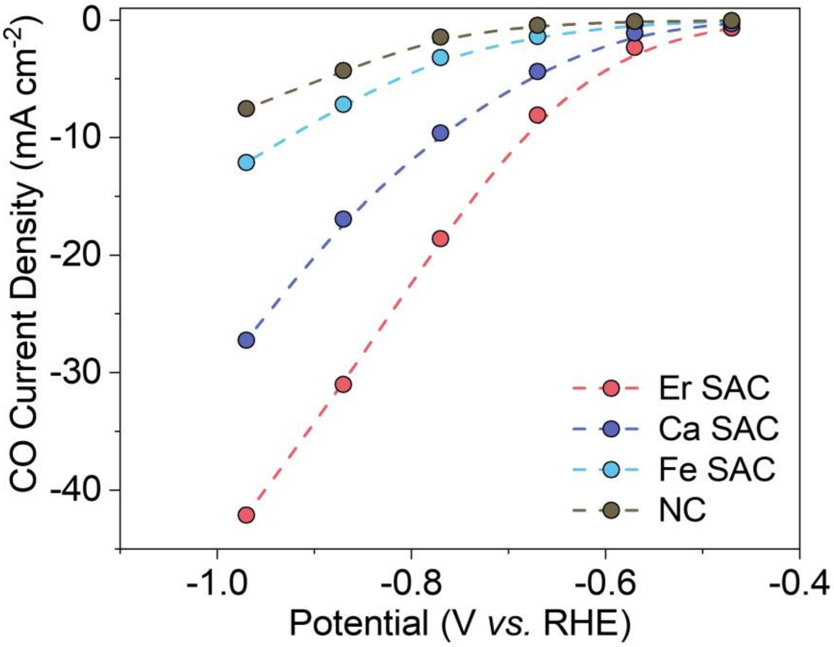

Supplementary Fig. 35 | CO partial current density over different catalysts in pure CO_2 saturated 0.5 M $KHCO_3$ solution in H-cell (without iR-correction).

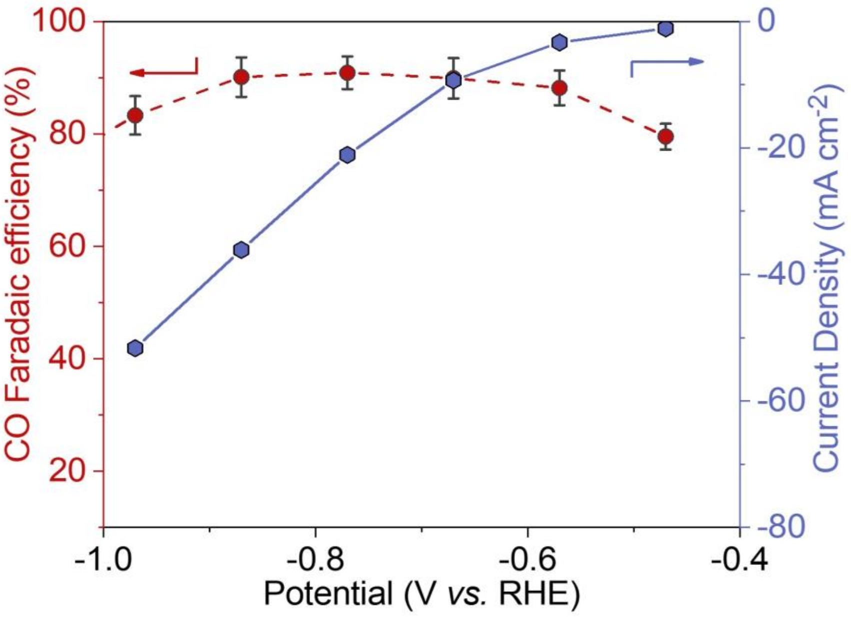

Supplementary Fig. 36 | FE_{CO} and total current density of Er SAC at different potentials in 30% CO_2 concentration saturated 0.5 M $KHCO_3$ solution in H-cell potentials (without iR-correction). The error bars correspond to the standard deviations of measurements over three separately prepared samples under the same testing conditions.

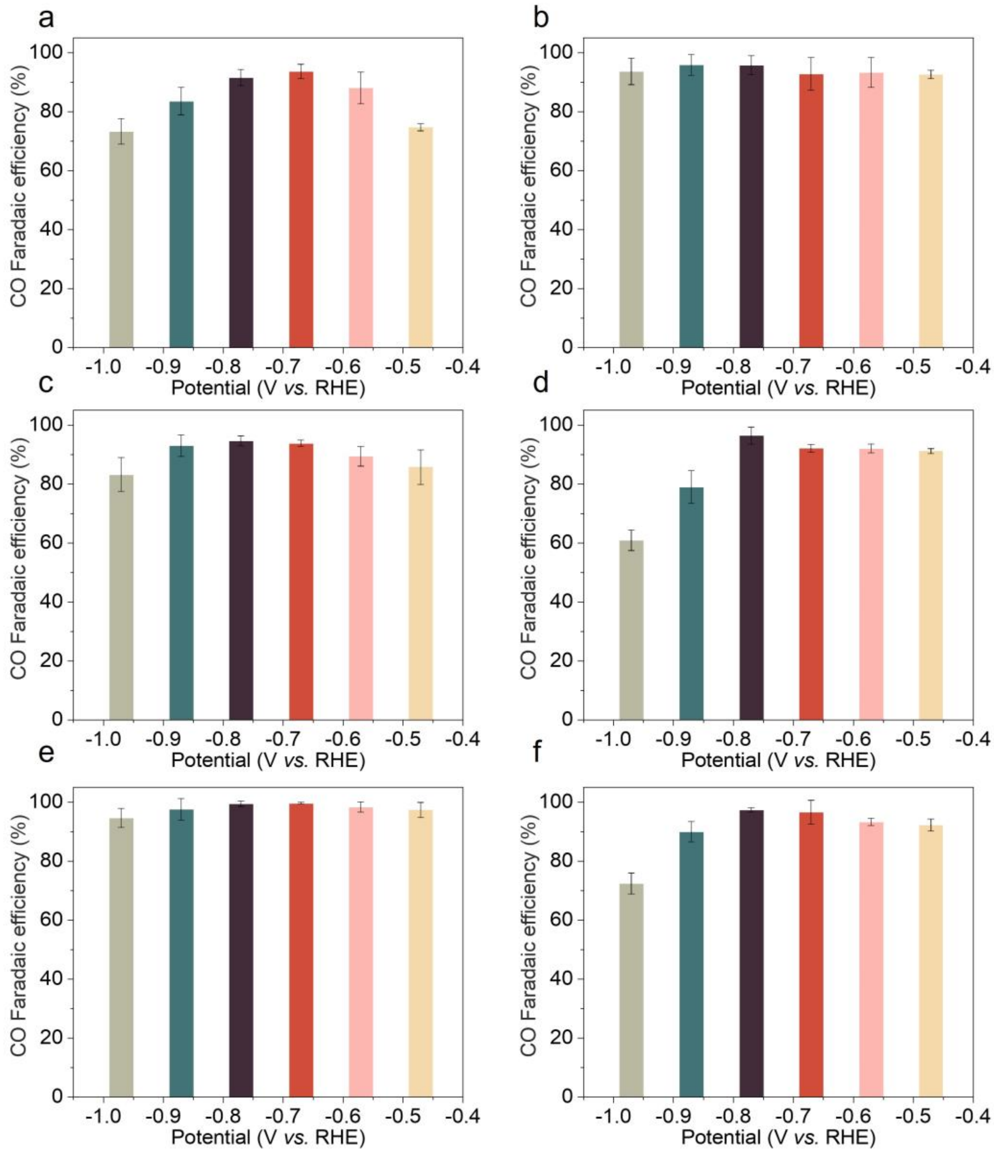

Supplementary Fig. 37 | FE_{CO} at different potentials in H-cell with pure CO_2 saturated 0.5 M $KHCO_3$ solution (without iR-correction). **b** FE_{CO} at. **a** La SAC. **b** Ce SAC. **c** Pr SAC. **d** Nd SAC. **e** Sm SAC. **f** Eu SAC. The error bars correspond to the standard deviations of measurements over three separately prepared samples under the same testing conditions.

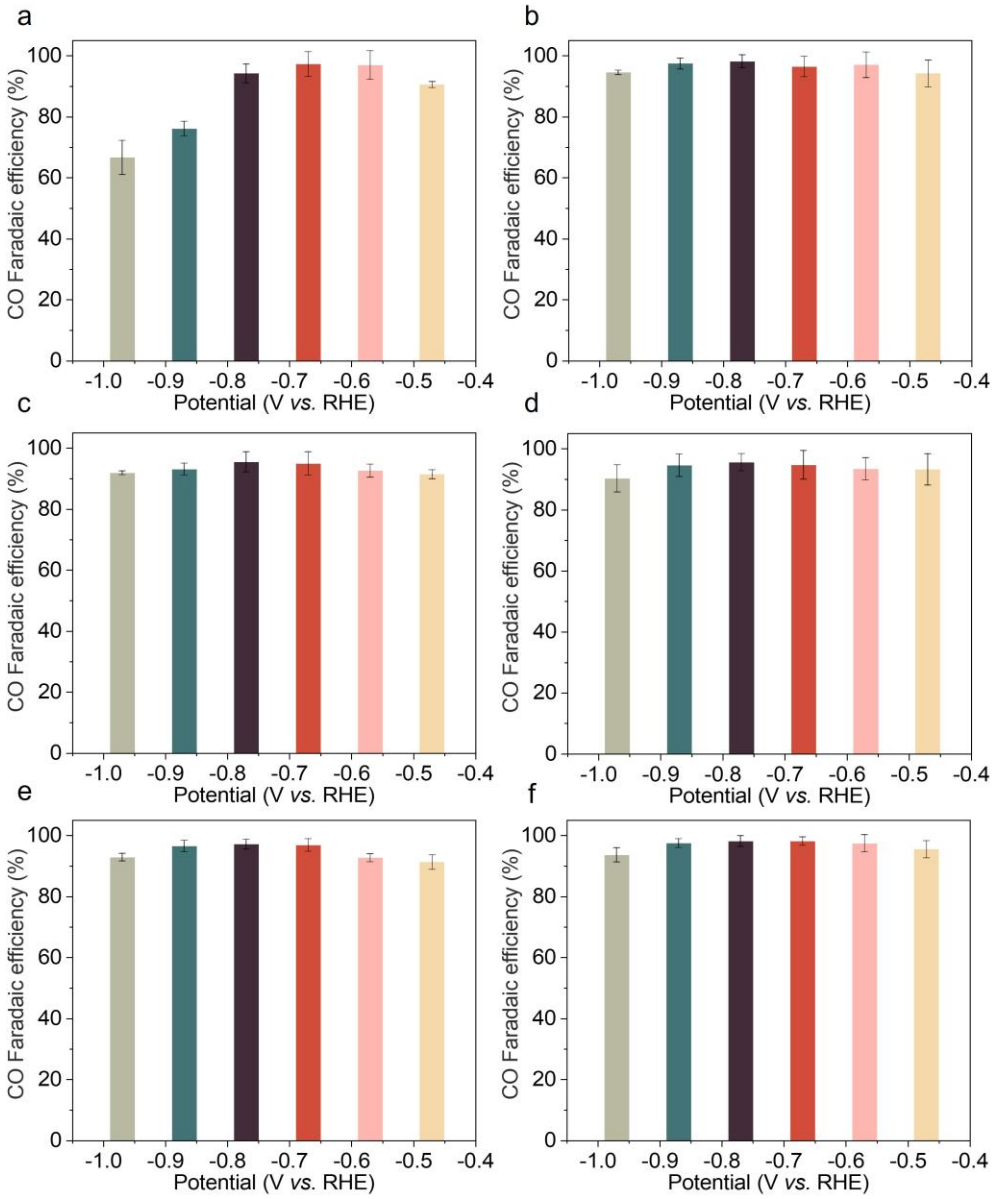

Supplementary Fig. 38 | FE_{CO} at different potentials in H-cell under with pure CO_2 saturated 0.5 M $KHCO_3$ solution (without iR-correction). **a** Gd SAC. **b** Tb SAC. **c** Dy SAC. **d** Ho SAC. **e** Er SAC. **f** Tm SAC. The error bars correspond to the standard deviations of measurements over three separately prepared samples under the same testing conditions.

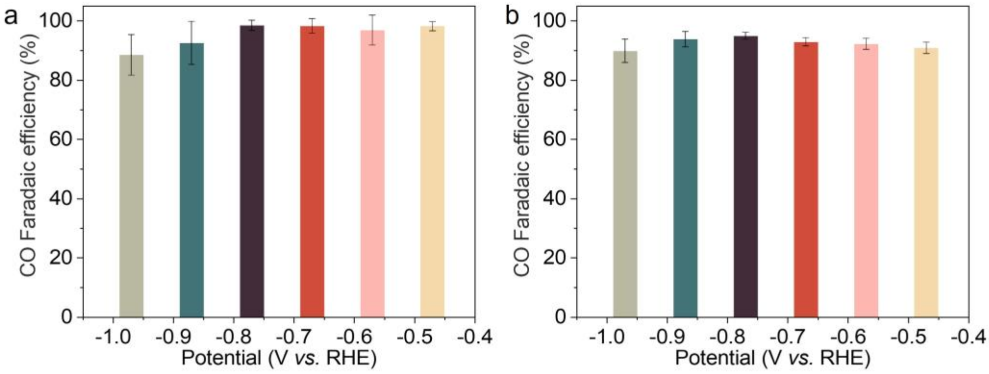

Supplementary Fig. 39 | FE_{CO} at different potentials with pure CO_2 saturated 0.5 M $KHCO_3$ solution (without iR-correction). **a** Yb SAC. **b** Lu SAC. The error bars correspond to the standard deviations of measurements over three separately prepared samples under the same testing conditions.

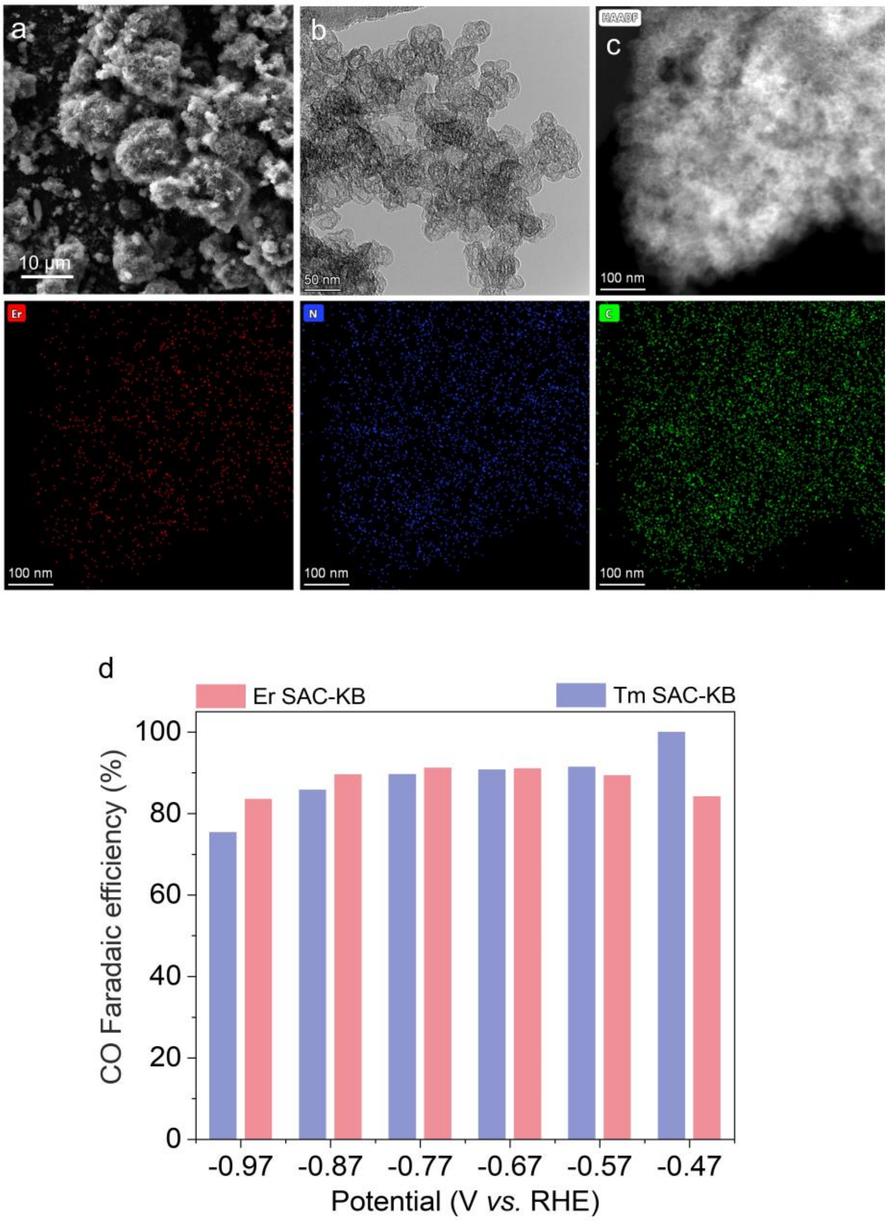

Supplementary Fig. 40|. a SEM, **b** TEM, **c** EDS mapping images for Er SAC with the substrate of Ketjen black (KB) substrate. **d** FE_{CO} at different potentials for representative Er and Tm SAC on KB substrate in H-cell with pure CO_2 saturated 0.5 M $KHCO_3$ solution (without iR-correction). This result demonstrates that the CO_2RR performance of Ln SACs exhibits a high independence of conductive substrate. The error bars correspond to the standard deviations of measurements over three separately prepared samples under the same testing conditions.

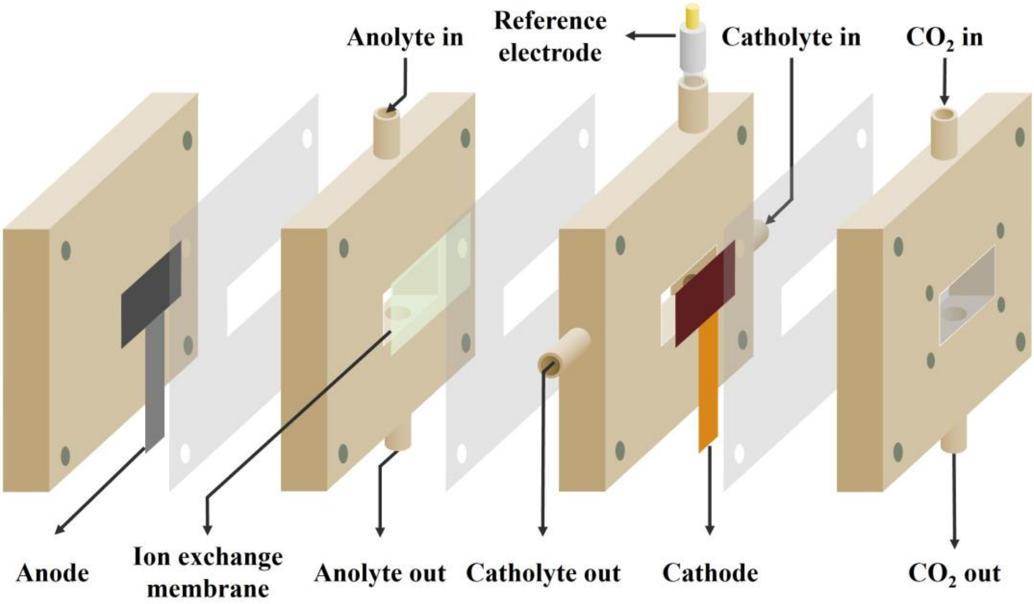

Supplementary Fig. 41 | Schematic representation of the flow cell.

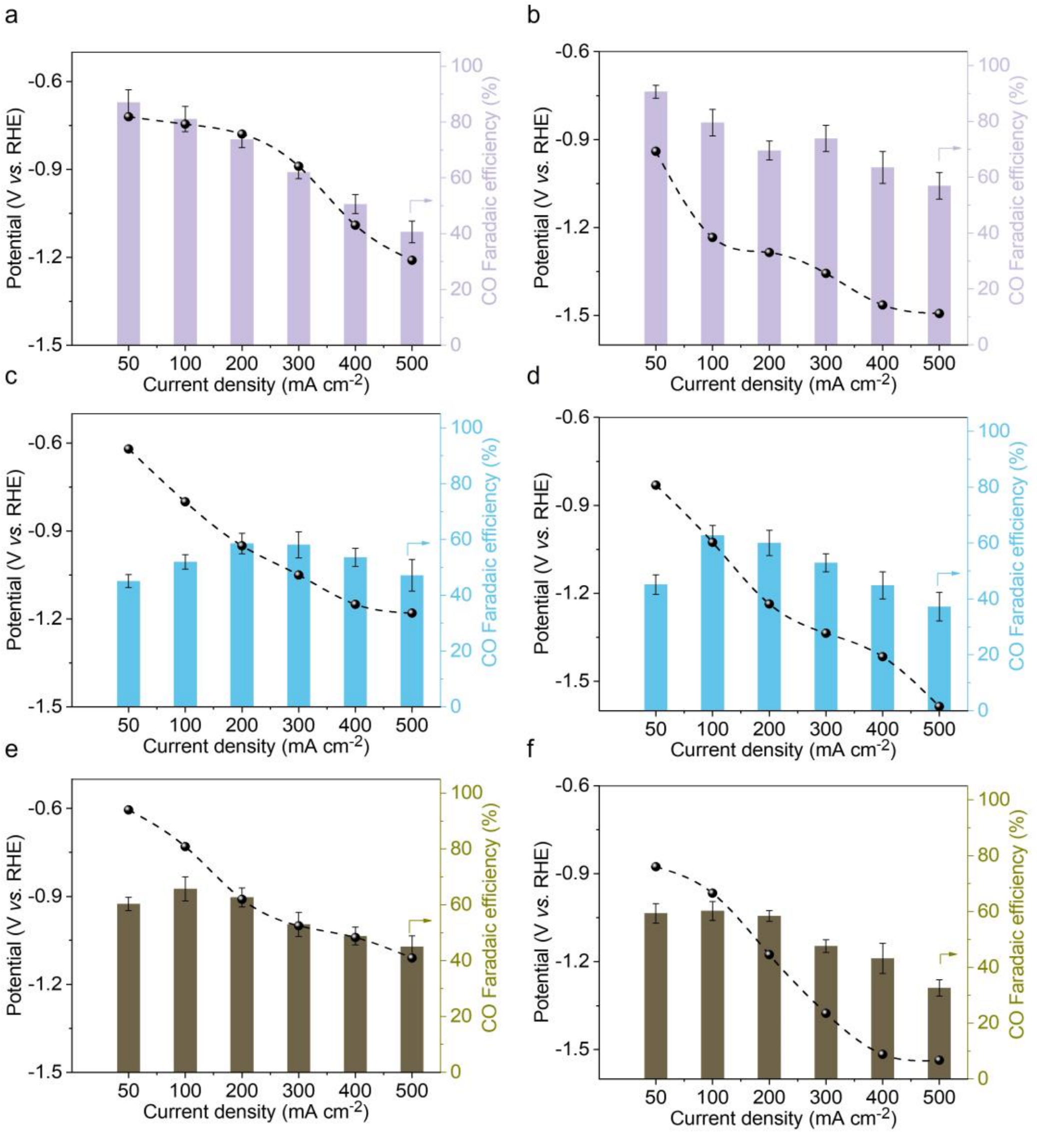

Supplementary Fig. 42 | Potentials and J_{CO} of catalysts at different current densities in flow cell (potential with 95% iR-correction, Resistance = 2.4 ± 0.3 Ω and 1.8 ± 0.2 Ω for neutral and acidic electrolyte, respectively). **a** Ca SAC with 1 M $KHCO_3$ solution. **b** Ca SAC with 1 M KCl (pH=1). **c** Fe SAC with 1 M $KHCO_3$ solution. **d** Fe with 1 M KCl (pH=1). **e** NC with 1 M $KHCO_3$ solution. **f** NC with 1 M KCl (pH=1). The error bars correspond to the standard deviations of measurements over three separately prepared samples under the same testing conditions.

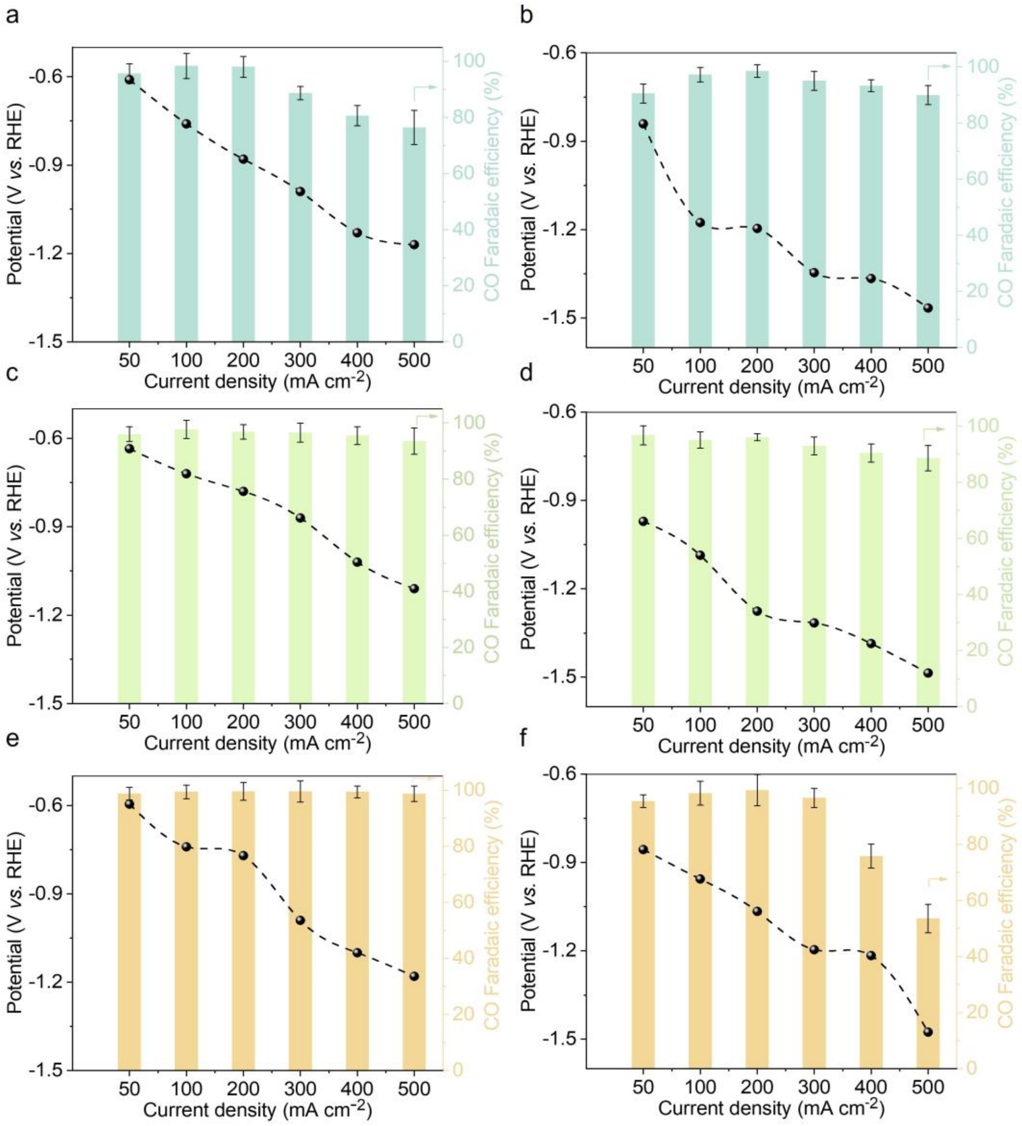

Supplementary Fig. 43 | Potentials and J_{CO} for representative Ln SACs at different current densities in flow cell (potential with 95% iR-correction, Resistance = 2.4 ± 0.3 Ω and 1.8 ± 0.2 Ω for neutral and acidic electrolyte, respectively). **a** Tm SAC with 1 M $KHCO_3$ solution. **b** Tm SAC with 1 M KCl (pH=1). **c** Sm SAC with 1 M $KHCO_3$ solution. **d** Sm with 1 M KCl (pH=1). **e** Lu with 1 M $KHCO_3$ solution. **f** Lu SAC with 1 M KCl (pH=1). The error bars correspond to the standard deviations of measurements over three separately prepared samples under the same testing conditions.

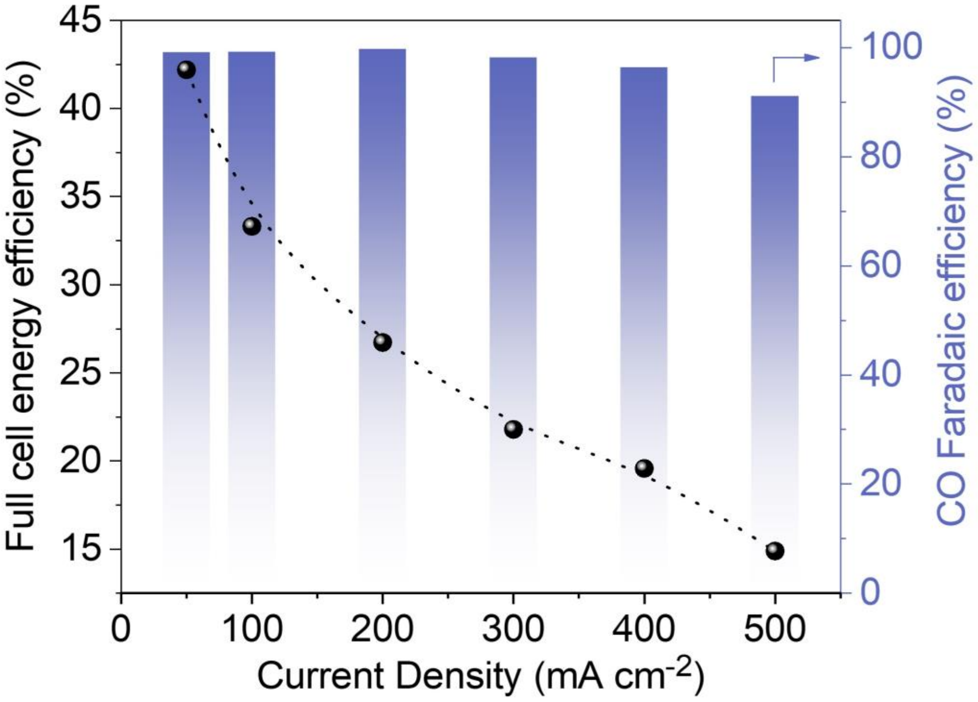

Supplementary Fig. 44 | Full cell energy efficiency for Er SAC in flow cell with 1 M KCl (pH=1).

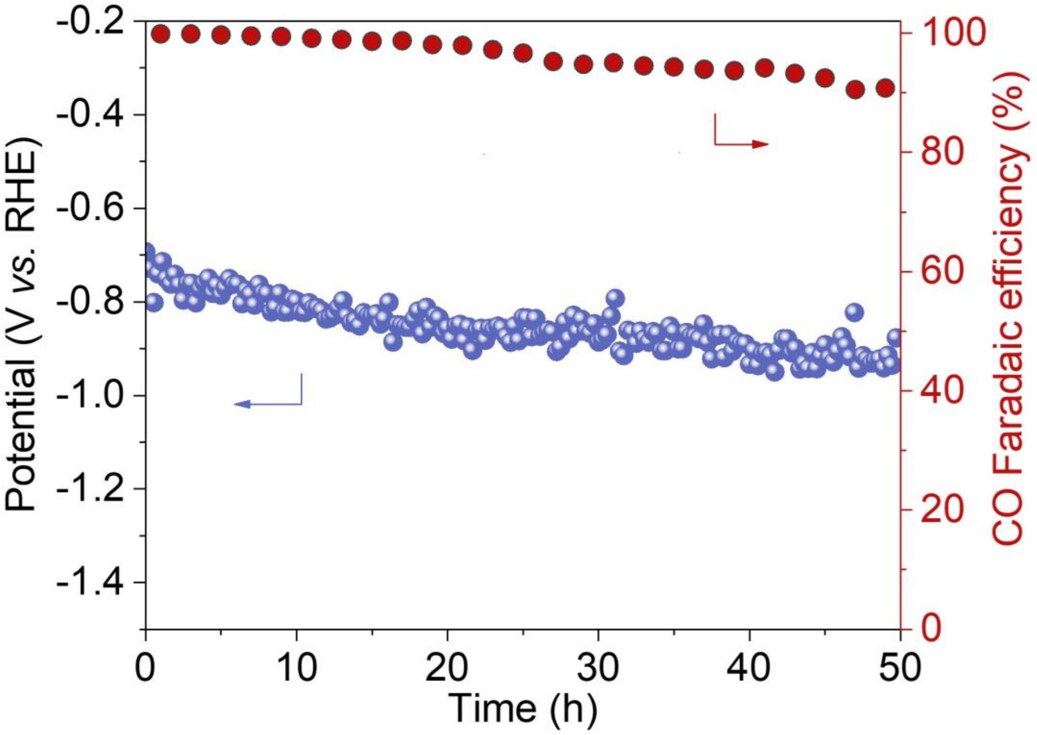

Supplementary Fig. 45 | Stability of Er SAC at a current density of −100 mA cm^{-2} in flow cell with CO_2 saturated 1 M $KHCO_3$ solution (potential with 95% iR-correction, Resistance = 2.4 ± 0.3 Ω and 1.8 ± 0.2 Ω for neutral and acidic electrolyte, respectively).

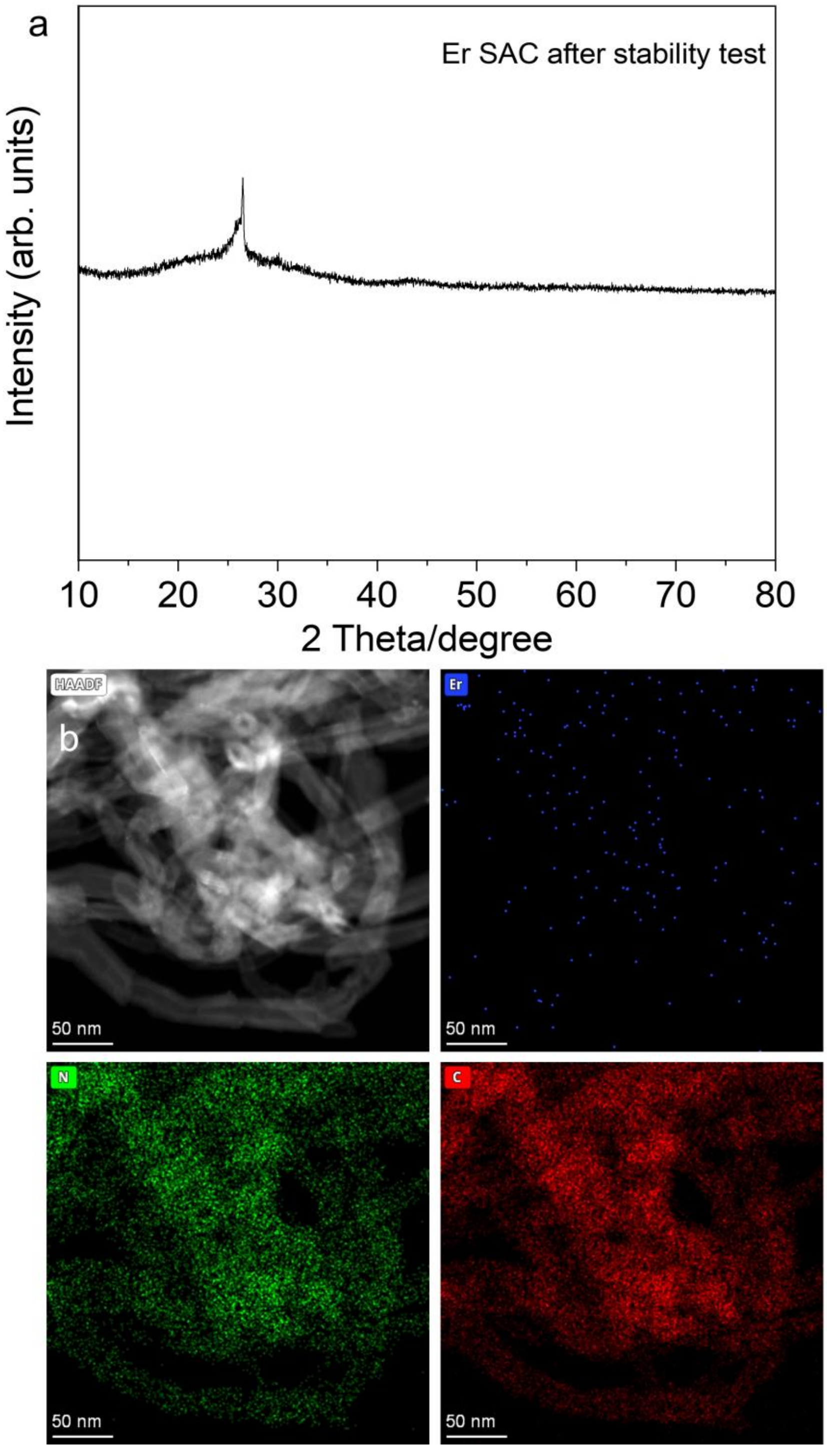

Supplementary Fig. 46 | a XRD pattern of Er SAC after stability test. **b** Dark-field scanning transmission electron microscopy and EDS mapping image of Er SAC after stability test.

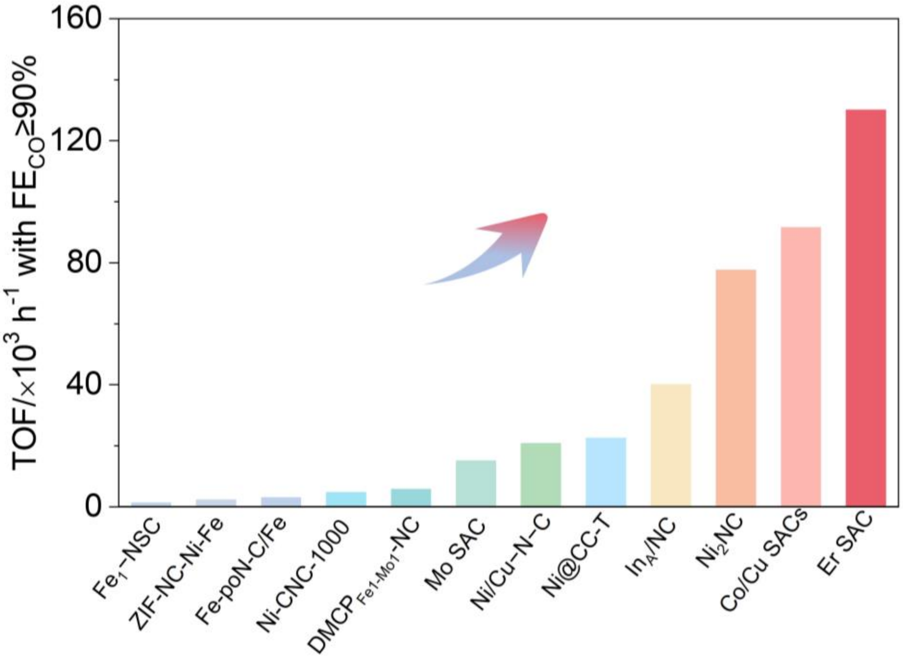

Supplementary Fig. 47 | Performance comparison of the state-of-art SACs for CO_2RR to CO.[1-11]

Supplementary Table 1 | Metal contents of Er, Fe and Ca SACs from ICP-OES.

	Er SAC	Ca SAC	Fe SAC
**Metal content (wt%)**	2.17	0.53	0.72

Supplementary Table 2 | EXAFS data fitting results of SACs

Sample	shell	CN	R(Å)	σ^2	ΔE_0	R factor
La SAC	La-N	10.02	2.57	0.0090	5.39±1.59	0.020
Ce SAC	Ce-N	1.4	2.17	0.0014	2.94±3.32	0.020
	Ce-N	6.1	2.39	0.0080		
Pr SAC	Pr-N	8.84	2.55	0.009	1.50±1.16	0.008
Nd SAC	Nd-N	8.28	2.52	0.0050	1.45±1.59	0.011
Sm SAC	Sm-N	9.39	2.47	0.0060	0.42±2.82	0.011
Eu SAC	Eu-N	9.24	2.44	0.0061	0.74±1.42	0.017
Gd SAC	Gd-N	9.21	2.44	0.007	0.17±1.03	0.006
Tb SAC	Tb-N	9.34	2.43	0.0070	0.06±1.35	0.015
Dy SAC	Dy-N	9.19	2.42	0.0070	0.91±1.61	0.009
Ho SAC	Ho-N	9.35	2.40	0.0093	0.04±1.31	0.139
Er SAC	Er-N	6.25	2.44	0.0038	4.00±1.89	0.018
Tm SAC	Tm-N	9.03	2.33	0.007	2.07±1.03	0.014
Yb SAC	Yb-N	9.31	2.33	0.008	-0.13±0.91	0.019
Lu SAC	Lu-N	9.16	2.33	0.007	2.24±1.11	0.011
Ca SAC	Ca-N	4.4	2.42	0.003	-0.05±0.02	0.016
Fe SAC	Fe-N	3.72	2.17	0.006	-2.34±1.62	0.003

CN: coordination numbers; R: bond distance; σ^2: Debye-Waller factors; ΔE_0: the inner potential correction. R factor: goodness of fit. S_0^2 was set to 0.87.

Supplementary Table 3 | Summary of detected substances and corresponding band positions for operando ATR-IR.

**Assignment**	**Band center (cm^{-1})**	**Reference**
$*CO_L$ stretching	~1890-1945	ACS Energy Lett. 2019, 4, 1778−1783<br>Angew. Chem. Int. Ed. 2020, 59, 12664–12668<br>Angew. Chem. Int. Ed. 2021, 60, 1–6
H−O−H bending	~1580-1650	ACS Cent. Sci. 2016, 2, 522−528<br>J. Am. Chem. Soc. 2020, 142, 11750−11762<br>ACS Energy Lett. 2019, 4, 1778−1783
CO_3^{2-}	~1400-1420	ACS Catal. 2020, 10, 8049−8057<br>J. Am. Chem. Soc. 2021, 143, 18233−18241<br>ACS Energy Lett. 2019, 4, 682−689
C−O (*COOH) stretching	~1395-1420	ACS Catal. 2017, 7, 606−612<br>Adv. Funct. Mater. 2021, 2104243<br>Angew. Chem. Int. Ed. 2022, 61, e202113918
C−OH (*COOH) deformation	~1288	ACS Catal. 2017, 7, 606−612<br>ACS Energy Lett. 2019, 4, 682−689
Si&HCO_3^- stretching	~1210-1250	J. Phys. Chem. C 2019, 123, 5951−5963<br>J. Am. Chem. Soc. 2020, 142, 11750−11762<br>ACS Nano 2022, 16, 2110−2119

Supplementary Table 4 | The full-cell energy efficiency (EE) of CO_2 to CO for Er SAC in flow-cell with 1 M KCl (pH=1) and 1 M $KHCO_3$ solution. The voltage is not iR corrected.

**Acidic electrolyte (Flow cell)**				**Neutral electrolyte (Flow cell)**			
**Current density (mA cm^{-2})**	**Voltage (V)**	**FE_{CO} (%)**	**EE (%)**	**Current density (mA cm^{-2})**	**Voltage (V)**	**FE_{CO} (%)**	**EE (%)**
50	3.15	99.2	42.2	50	4.21	99.8	31.7
100	3.99	99.2	33.3	100	6.05	94.6	21.0
200	5.01	99.7	26.7	200	9.72	93.9	13.0
300	6.04	98.2	21.8	300	12.71	93.2	9.8
400	6.60	96.4	19.6	400	15.51	90.0	7.8
500	8.21	91.1	14.9	500	16.70	90.0	7.2

Supplementary Table 5 | The full-cell energy efficiency (EE) of CO_2 to CO for Er SAC in MEA with 0.5 M K_2SO_4 (pH adjusted to 1.0 with sulfuric acid) electrolyte. The voltage is without iR corrected.

**Acidic electrolyte (MEA)**			
**Current density ($mA\ cm^{-2}$)**	**Voltage (V)**	**FE_{CO} (%)**	**EE (%)**
50	3.08	99.3	43.2
100	3.43	99.1	38.7
200	3.76	97.3	34.7
300	3.98	96.4	32.5
400	4.17	93.1	29.9
500	4.42	90.2	27.3

Supplementary Table 6 | The single pass efficiency (SPCE) of CO_2 to CO for Er SAC at different CO_2 flow rates with 1 M KCl (pH=1) electrolyte in flow cell. The error bars correspond to the standard deviations of measurements over three separately prepared samples under the same testing conditions. The error bar is calculated with SPCE%, rather than the total.

**Flow rate (20 sccm)**				**Flow rate (10 sccm)**			
**j (mA cm^{-2})**	**FE_{CO} (%)**	**SPCE (%)**	**Error (% of SPCE)**	**j (mA cm^{-2})**	**FE_{CO} (%)**	**SPCE (%)**	**Error (% of SPCE)**
100	99.21	1.7	3.5	100	91.98	3.4	2.3
200	99.72	3.7	3.7	200	98.29	7.4	1.6

**Flow rate (5 sccm)**				**Flow rate (2 sccm)**			
**j (mA cm^{-2})**	**FE_{CO} (%)**	**SPCE (%)**	**Error (% of SPCE)**	**j (mA cm^{-2})**	**FE_{CO} (%)**	**SPCE (%)**	**Error (% of SPCE)**
100	98.95	7.4	3.1	100	97.55	18.2	3.6
200	98.23	14.7	2.4	200	98.18	36.7	2.8

**Flow rate (1 sccm)**			
**j (mA cm^{-2})**	**FE_{CO} (%)**	**SPCE (%)**	**Error (% of SPCE)**
100	96.41	36.0	1.8
200	94.13	70.4	3.1

Supplementary Table 7 | Faradaic efficiency of products at different potentials in H-cell with pure CO_2 saturated 0.5 M $KHCO_3$ solution. The error bars correspond to the standard deviations of measurements over three separately prepared samples under the same testing conditions.

	La SAC				Ce SAC			
Potential (V vs.RHE)	FE_{CO} (%)	Error bar (%)	FE_{H2} (%)	Error bar (%)	FE_{CO} (%)	Error bar (%)	FE_{H2} (%)	Error bar (%)
-0.47 V	74.6	1.2	25.4	3.4	92.5	1.5	7.5	1.1
-0.57 V	87.9	5.4	12.1	2.3	93.2	5.0	6.8	0.9
-0.67 V	93.5	2.4	6.5	1.3	92.7	5.5	7.3	1.2
-0.77 V	91.4	2.8	8.6	1.1	95.6	3.2	4.4	1.5
-0.87 V	83.5	4.6	16.5	1.9	95.7	3.5	4.3	2.3
-0.97 V	73.2	4.3	26.8	1.7	93.5	4.4	6.5	1.4
	Pr SAC				Nd SAC			
Potential (V vs.RHE)	FE_{CO} (%)	Error bar (%)	FE_{H2} (%)	Error bar (%)	FE_{CO} (%)	Error bar (%)	FE_{H2} (%)	Error bar (%)
-0.47 V	85.6	5.9	14.4	1.2	91.0	0.8	9.0	1.2
-0.57 V	89.3	3.3	10.7	1.1	92.0	1.5	8.0	1.9
-0.67 V	93.7	1.1	6.3	1.9	92.0	1.3	8.0	2.2
-0.77 V	94.5	1.7	5.5	2.3	96.3	2.9	3.7	2.8
-0.87 V	92.9	3.7	7.1	1.5	78.9	5.6	21.1	4.3
-0.97 V	83.1	5.7	16.9	3.1	60.8	3.5	39.2	5.7
	Sm SAC				Eu SAC			
Potential (V vs.RHE)	FE_{CO} (%)	Error bar (%)	FE_{H2} (%)	Error bar (%)	FE_{CO} (%)	Error bar (%)	FE_{H2} (%)	Error bar (%)
-0.47 V	97.2	2.6	2.8	1.2	92.1	2.1	7.9	2.1
-0.57 V	98.3	1.8	1.7	1.9	93.2	1.2	6.8	2.8
-0.67 V	99.5	0.3	0.5	0.5	96.5	4.1	3.5	1.2
-0.77 V	99.3	0.9	0.7	0.7	97.3	0.8	2.7	1.4
-0.87 V	97.5	3.6	2.5	0.6	89.9	3.5	10.1	3.5
-0.97 V	94.6	3.2	5.4	2.1	72.3	3.6	27.7	4.8
	Gd SAC				Tb SAC			
Potential (V vs.RHE)	FE_{CO} (%)	Error bar (%)	FE_{H2} (%)	Error bar (%)	FE_{CO} (%)	Error bar (%)	FE_{H2} (%)	Error bar (%)
-0.47 V	90.5	1.0	9.5	2.7	94.2	4.4	5.8	2.6
-0.57 V	97.0	4.7	3.0	1.8	97.0	4.2	3.0	1.7
-0.67 V	97.3	4.1	2.7	1.5	96.4	3.4	3.6	2.5
-0.77 V	94.2	3.1	5.8	2.8	98.2	2.1	1.8	1.3
-0.87 V	76.1	2.4	23.9	5.6	97.4	1.8	2.6	1.8
-0.97 V	66.7	5.6	33.3	6.2	94.6	0.7	5.4	2.3

	Dy SAC				Ho SAC			
Potential (V vs.RHE)	FE_{CO} (%)	Error bar (%)	FE_{H2} (%)	Error bar (%)	FE_{CO} (%)	Error bar (%)	FE_{H2} (%)	Error bar (%)
-0.47 V	91.4	1.5	8.6	2.9	93.2	5.1	6.8	1.1
-0.57 V	92.6	2.1	7.4	2.4	93.4	3.6	6.6	1.3
-0.67 V	95.0	3.8	5.0	1.5	94.7	4.7	5.3	1.7
-0.77 V	95.5	3.3	4.5	1.3	95.6	2.8	4.4	0.9
-0.87 V	93.1	1.9	6.9	2.4	94.6	3.7	5.4	1.4
-0.97 V	91.9	0.6	8.1	3.7	90.3	4.5	9.7	3.2
	Er SAC				Tm SAC			
Potential (V vs.RHE)	FE_{CO} (%)	Error bar (%)	FE_{H2} (%)	Error bar (%)	FE_{CO} (%)	Error bar (%)	FE_{H2} (%)	Error bar (%)
-0.47 V	91.3	2.4	8.7	1.7	95.5	2.8	4.5	1.3
-0.57 V	92.7	1.3	7.3	1.9	97.4	2.8	2.6	1.1
-0.67 V	96.9	2.1	3.1	1.1	98.2	1.4	1.8	0.7
-0.77 V	97.2	1.6	2.8	0.8	98.2	1.8	1.8	0.9
-0.87 V	96.5	1.9	3.5	1.3	97.5	1.4	2.5	1.6
-0.97 V	92.9	1.2	7.1	2.5	93.7	2.3	6.3	2.7
	Yb SAC				Lu SAC			
Potential (V vs.RHE)	FE_{CO} (%)	Error bar (%)	FE_{H2} (%)	Error bar (%)	FE_{CO} (%)	Error bar (%)	FE_{H2} (%)	Error bar (%)
-0.47 V	98.1	1.5	1.9	0.7	90.9	1.9	9.1	1.3
-0.57 V	96.9	5.1	3.1	1.3	92.2	1.9	7.8	1.1
-0.67 V	98.3	2.5	1.7	1.1	92.9	1.4	7.1	1.0
-0.77 V	98.4	1.7	1.6	0.8	95.0	1.2	5.0	0.6
-0.87 V	92.5	7.3	7.5	1.8	93.8	2.6	6.2	0.8
-0.97 V	88.5	6.8	11.5	3.9	89.8	4.0	10.2	2.1
	Ca SAC				Fe SAC			
Potential (V vs.RHE)	FE_{CO} (%)	Error bar (%)	FE_{H2} (%)	Error bar (%)	FE_{CO} (%)	Error bar (%)	FE_{H2} (%)	Error bar (%)
-0.47 V	30.2	5.1	69.8	5.5	23.3	3.6	76.7	5.4
-0.57 V	42.2	8.8	57.8	5.6	42.9	4.3	57.1	4.2
-0.67 V	71.2	2.8	28.8	3.5	62.3	2.3	37.7	4.1
-0.77 V	79.8	4.4	20.2	3.4	63.9	4.1	36.1	3.7
-0.87 V	82.1	6.5	17.9	2.8	68.0	2.6	32.0	2.6
-0.97 V	78.6	2.6	21.4	3.0	60.6	4.5	39.4	4.9

	NC				Er SAC KB		Tm SAC KB	
Potential (V vs.RHE)	FE_{CO} (%)	Error bar (%)	FE_{H2} (%)	Error bar (%)	FE_{CO} (%)	FE_{H2} (%)	FE_{CO} (%)	FE_{H2} (%)
-0.47 V	51.5	3.5	48.5	4.7	84.2	15.8	99.6	0.4
-0.57 V	56.1	1.0	43.9	5.3	89.3	10.7	91.5	8.5
-0.67 V	60.7	3.1	39.3	3.9	91.1	8.9	90.8	9.2
-0.77 V	65.9	2.3	34.1	3.9	91.3	8.7	89.6	10.4
-0.87 V	56.1	5.6	43.9	4.7	89.6	10.4	85.8	14.2
-0.97 V	43.2	7.3	56.8	6.4	83.6	16.4	75.4	24.6

Supplementary Table 8 | Faradaic efficiency of products at different current densities in flow cell with 1 M $KHCO_3$ solution. The error bars correspond to the standard deviations of measurements over three separately prepared samples under the same testing conditions.

	Sm SAC				Tm SAC			
Current density (mA cm^{-2})	FE_{CO} (%)	Error bar (%)	FE_{H2} (%)	Error bar (%)	FE_{CO} (%)	Error bar (%)	FE_{H2} (%)	Error bar (%)
50	96.0	2.6	4.0	0.9	95.6	3.5	4.4	1.1
100	97.7	3.2	2.3	0.6	98.3	4.5	1.7	1.7
200	96.8	2.6	3.2	0.6	98.0	3.7	2.0	0.8
300	96.5	3.4	3.5	1.1	88.7	2.4	11.3	1.5
400	95.5	3.2	4.5	1.5	80.6	3.6	19.4	2.7
500	93.6	4.7	6.4	2.4	76.4	6.1	23.6	3.9
	Lu SAC				Ca SAC			
Current density (mA cm^{-2})	FE_{CO} (%)	Error bar (%)	FE_{H2} (%)	Error bar (%)	FE_{CO} (%)	Error bar (%)	FE_{H2} (%)	Error bar (%)
50	98.8	2.3	1.2	0.7	87.0	4.5	13.0	1.3
100	99.4	2.4	0.6	0.3	81.1	4.5	18.9	1.6
200	99.6	3.2	0.4	0.1	73.7	2.9	26.3	2.8
300	99.6	3.8	0.4	0.2	62.0	2.3	38.0	3.5
400	99.4	2.1	0.6	0.2	50.6	3.4	49.4	5.9
500	98.7	2.8	1.3	0.7	40.6	3.9	59.4	6.3
	Fe SAC				NC			
Current density (mA cm^{-2})	FE_{CO} (%)	Error bar (%)	FE_{H2} (%)	Error bar (%)	FE_{CO} (%)	Error bar (%)	FE_{H2} (%)	Error bar (%)
50	45.1	2.3	54.9	2.8	60.3	2.4	39.7	4.7
100	52.0	2.6	48.0	4.3	65.7	4.3	34.3	3.8
200	58.5	3.7	41.5	4.7	62.7	3.4	37.3	3.6
300	58.1	4.7	41.9	5.6	52.9	4.3	47.1	3.8
400	53.6	3.2	46.4	6.4	48.8	3.2	51.2	5.1
500	47.1	5.7	52.9	5.6	45.0	3.8	55.0	5.3

Supplementary Table 9 | Supplementary Table 9 | Faradaic efficiency of products at different current densities in flow cell with 1 M KCl (pH=1). The error bars correspond to the standard deviations of measurements over three separately prepared samples under the same testing conditions.

	Sm SAC				Tm SAC			
Current density (mA cm^{-2})	FE_{CO} (%)	Error bar (%)	FE_{H2} (%)	Error bar (%)	FE_{CO} (%)	Error bar (%)	FE_{H2} (%)	Error bar (%)
50	96.7	3.4	3.3	0.6	90.5	3.4	9.5	3.1
100	95.1	2.9	4.9	0.7	97.2	2.6	2.8	0.4
200	96.1	1.3	3.9	1.1	98.5	2.3	1.5	0.7
300	93.0	3.2	7.0	2.8	95.1	3.4	4.9	1.6
400	90.4	3.2	9.6	2.5	93.3	2.1	6.7	1.7
500	88.6	4.5	11.4	3.2	89.9	3.4	10.1	2.4
	Lu SAC				Ca SAC			
Current density (mA cm^{-2})	FE_{CO} (%)	Error bar (%)	FE_{H2} (%)	Error bar (%)	FE_{CO} (%)	Error bar (%)	FE_{H2} (%)	Error bar (%)
50	95.4	2.3	4.6	1.3	90.6	2.3	9.4	3.2
100	98.2	4.3	1.8	0.2	79.5	4.7	20.5	2.5
200	99.3	5.6	0.7	0.1	69.5	3.4	30.5	3.0
300	96.5	3.4	3.5	0.7	73.9	4.7	26.1	2.9
400	75.8	4.3	24.2	2.6	63.5	5.7	36.5	3.2
500	53.5	5.1	46.5	4.9	56.9	4.8	43.1	6.6
	Fe SAC				NC			
Current density (mA cm^{-2})	FE_{CO} (%)	Error bar (%)	FE_{H2} (%)	Error bar (%)	FE_{CO} (%)	Error bar (%)	FE_{H2} (%)	Error bar (%)
50	45.2	3.5	54.8	3.7	59.3	3.5	40.7	3.6
100	62.7	3.6	37.3	5.4	60.2	3.4	39.8	2.6
200	60.0	4.5	40.0	3.3	58.4	1.9	41.6	3.5
300	53.0	3.2	47.0	2.1	47.6	2.3	52.4	3.3
400	44.9	4.8	55.1	2.3	43.2	5.4	56.8	4.4
500	37.2	5.1	62.8	4.7	32.6	2.9	67.4	4.8

Supplementary Table 10 | Faradaic efficiency of products for Er SAC in MEA with 0.5 M K_2SO_4 (pH adjusted to 1.0 with sulfuric acid) electrolyte.

	Er SAC			
Current density ($mA\ cm^{-2}$)	FE_{CO} (%)	Error bar (%)	FE_{H2} (%)	Error bar (%)
50	99.3	0.5	0.7	0.2
100	99.1	0.3	0.9	0.2
200	97.3	1.2	2.7	0.4
300	96.4	1.7	3.6	0.7
400	93.1	2.4	6.9	1.1
500	90.2	2.3	9.8	1.7

Supplementary Table 11 | Faradaic efficiency of products for Er SAC at different CO_2 flow rates with 1 M KCl (pH=1) electrolyte in flow cell. The error bars correspond to the standard deviations of measurements over three separately prepared samples under the same testing conditions.

Current density (mA cm^{-2})	20 sccm				10 sccm			
	FE_{CO} (%)	Error bar (%)	FE_{H2} (%)	Error bar (%)	FE_{CO} (%)	Error bar (%)	FE_{H2} (%)	Error bar (%)
100	99.2	3.5	0.8	0.4	92.0	2.3	8.0	1.1
200	99.7	3.7	0.3	0.1	98.3	1.6	1.7	0.7

Current density (mA cm^{-2})	5 sccm				2 sccm			
	FE_{CO} (%)	Error bar (%)	FE_{H2} (%)	Error bar (%)	FE_{CO} (%)	Error bar (%)	FE_{H2} (%)	Error bar (%)
100	98.9	3.1	1.1	0.2	97.6	3.6	2.4	0.7
200	98.2	2.4	1.8	0.6	98.2	2.8	1.8	0.4

Current density (mA cm^{-2})	1 sccm			
	FE_{CO} (%)	Error bar (%)	FE_{H2} (%)	Error bar (%)
100	96.4	1.8	3.6	0.8
200	94.1	3.1	5.9	1.6

Supplementary Table 12 | Comparison of full-cell energy efficiency of various catalysts during CO_2 electroreduction in acidic electrolyte.

**Catalysts**	**Current density (mA/cm^{-2})**	**Full-cell energy efficiency (%)**	**Ref.**
Er SAC-Flow cell	50	42.2	This work
Er SAC-MEA	50	43.2	This work
Er SAC-MEA	100	38.7	This work
Er SAC-MEA	200	34.7	This work
CG-medium Cu[12]	100	~28	Nat. Catal. 2023, 6, 763
PFSA modified Cu[13]	200	~25	Nat. Synth. 2023, 2, 403
NiNC-IMI[14]	200	~40	Nat. Chem. Eng. 2024, 1, 229
EC-Cu[15]	200	~30	Nat. Commun. 2023, 14, 2387
Co-CNTs-MW[16]	200	~54.1	Nat. Commun. 2023, 14, 1599
$Cu_{0.9}Zn_{0.1}$[17]	150	~31.2	Nat. Commun. 2023, 14, 1298
ER-CuNS[18]	800	~10	Nat. Commun. 2022, 13, 7596

Supplementary Table 13 | Comparison of single-pass carbon efficiency of various catalysts during CO_2 electroreduction in acidic electrolyte.

**Catalysts**	**Current density (mA/cm^{-2})**	**Single-pass carbon efficiency (%)**	**Ref.**
Er SAC	200	70.4	This work
CG-medium Cu[12]	100	~90	Nat. Catal. 2023, 6, 763
Pd-Cu[19]	500	~62	Nat. Catal. 2022, 5, 564
PFSA modified Cu[13]	200	~75	Nat. Synth. 2023, 2, 403
CoTAAPc@CNT-n[20]	256	~67.4	Nat. Synth. 2024, 3, 1231
NiNC-IMI[14]	200	~40	Nat. Chem. Eng. 2024, 1, 229
Cu-GDL[21]	500	~42	Nat. Commun. 2024, 15, 491
EC-Cu[15]	200	~70	Nat. Commun. 2023, 14, 2387
Sn(S)-H[22]	400	~36	Nat. Commun. 2023, 14, 2843
Co-CNTs-MW[16]	100	~40.4	Nat. Commun. 2023, 14, 1599
$Cu_{0.9}Zn_{0.1}$[17]	400	~31	Nat. Commun. 2023, 14, 1298
ER-CuNS[18]	800	~54	Nat. Commun. 2022, 13, 7596

Supplementary Table 14 | Comparison of CO_2-to-CO TOF of the state-of-art catalysts.

Catalysts	The Maximal TOF ($hour^{-1}$) under $FE_{CO} \geqslant 90\%$	Cell type	Ref.
Er SAC	130,000	Flow cell	This work
NiPc–OMe MDE[23]	43,200	Flow cell	Nat. Energy 2020, 5, 684
Ni_2NC[11]	77,500	Flow cell	Nat. Synth. 2022, 1, 719
CoCu-DASC[6]	91,458	Flow cell	Angew. Chem. Int. Ed. 2022, 61, e202212329
Er SAC	60,000	H-cell	This work
Fe^{3+}–N–C[24]	1,000	H-cell	Science 2019, 364, 1091
A-Ni-NSG[25]	14,800	H-cell	Nat. Energy 2018, 3, 140
Co-S_1N_3[26]	4,564	H-cell	Nat. Commun. 2024, 15, 416
Fe-poN-C/Fe[10]	2,890	H-cell	Nat. Commun. 2023, 14, 5108
Co-CNTs-MW[16]	25,896	H-cell	Nat. Commun. 2023, 14, 1599
TeN_2–CuN_3[27]	24,080	H-cell	Nat. Commun. 2023, 14, 6164
Ni@C_3N_4-CN[28]	22,000	H-cell	Nat. Commun. 2022, 13, 6082
NiFe-DASC[29]	15,055	H-cell	Nat. Commun. 2021, 12, 4088
Ni–N_2C_2[30]	3,903	H-cell	Nat. Commun. 2020, 11, 2256
(Cl, N)-Mn/G[31]	38,347	H-cell	Nat. Commun. 2019, 10, 2980
Ni-NCB[32]	36,000	H-cell	Joule 2019, 3, 265

Reference

1. Wang, T. et al. Single-Atom Anchored Curved Carbon Surface for Efficient CO2 Electro-Reduction with Nearly 100% CO Selectivity and Industrially-Relevant Current Density. *Advanced Materials* **35**, 2205553 (2023).
2. Zhang, L. et al. Atomically Dispersed Ni–Cu Catalysts for pH-Universal CO2 Electroreduction. *Advanced Materials* **35**, 2209590 (2023).
3. Cao, X. et al. Atomic Bridging Structure of Nickel–Nitrogen–Carbon for Highly Efficient Electrocatalytic Reduction of CO_2. *Angewandte Chemie International Edition* **61**, e202113918 (2021).
4. Chen, S. et al. Unveiling the Proton-Feeding Effect in Sulfur-Doped Fe−N−C Single-Atom Catalyst for Enhanced CO2 Electroreduction. *Angewandte Chemie International Edition* **61**, e202206233 (2022).
5. Li, Y. et al. Atomically Dispersed Dual-Metal Site Catalysts for Enhanced CO2 Reduction: Mechanistic Insight into Active Site Structures. *Angewandte Chemie International Edition* **n/a**, e202205632 (2022).
6. Yi, J.-d., Gao, X., Zhou, H., Chen, W. & Wu, Y. Design of Co-Cu Diatomic Site Catalysts for High-efficiency Synergistic CO2 Electroreduction at Industrial-level Current Density. *Angewandte Chemie International Edition* **61**, e202212329 (2022).
7. Ding, J. et al. Circumventing CO2 Reduction Scaling Relations Over the Heteronuclear Diatomic Catalytic Pair. *Journal of the American Chemical Society* **145**, 11829-11836 (2023).
8. Guo, W. et al. Atomic Indium Catalysts for Switching CO2 Electroreduction Products from Formate to CO. *Journal of the American Chemical Society* (2021).
9. Driscoll, D.M. et al. Observation of a promethium complex in solution. *Nature* **629**, 819-823 (2024).
10. Wang, C. et al. Combining Fe nanoparticles and pyrrole-type Fe-N4 sites on less-oxygenated carbon supports for electrochemical CO2 reduction. *Nature Communications* **14**, 5108 (2023).
11. Hao, Q. et al. Nickel dual-atom sites for electrochemical carbon dioxide reduction. *Nature Synthesis* **1**, 719-728 (2022).
12. Fan, M. et al. Cationic-group-functionalized electrocatalysts enable stable acidic CO2 electrolysis. *Nature Catalysis* **6**, 763-772 (2023).
13. Zhao, Y. et al. Conversion of CO2 to multicarbon products in strong acid by controlling the catalyst microenvironment. *Nature Synthesis* **2**, 403-412 (2023).
14. Brückner, S. et al. Design and diagnosis of high-performance CO2-to-CO electrolyzer cells. *Nature Chemical Engineering* **1**, 229-239 (2024).
15. Cao, Y. et al. Surface hydroxide promotes CO2 electrolysis to ethylene in acidic conditions. *Nature Communications* **14**, 2387 (2023).
16. Sun, J.W. et al. Scalable synthesis of coordinatively unsaturated metal-nitrogen sites for large-scale CO2 electrolysis. *Nature Communications* **14**, 1599 (2023).
17. Zhang, J. et al. Accelerating electrochemical CO2 reduction to multi-carbon products via asymmetric intermediate binding at confined nanointerfaces. *Nature Communications* **14**, 1298 (2023).
18. Ma, Z. et al. CO2 electroreduction to multicarbon products in strongly acidic electrolyte via synergistically modulating the local microenvironment. *Nature Communications* **13**, 7596 (2022).

19. Xie, Y. et al. High carbon utilization in CO2 reduction to multi-carbon products in acidic media. *Nature Catalysis* **5**, 564-570 (2022).
20. Zhang, Q. et al. A covalent molecular design enabling efficient CO2 reduction in strong acids. *Nature Synthesis* (2024).
21. Sun, M., Cheng, J. & Yamauchi, M. Gas diffusion enhanced electrode with ultrathin superhydrophobic macropore structure for acidic CO2 electroreduction. *Nature Communications* **15**, 491 (2024).
22. Shen, H. et al. Acidic CO2-to-HCOOH electrolysis with industrial-level current on phase engineered tin sulfide. *Nature Communications* **14**, 2843 (2023).
23. Zhang, X. et al. Molecular engineering of dispersed nickel phthalocyanines on carbon nanotubes for selective CO2 reduction. *Nature Energy* **5**, 684-692 (2020).
24. Gu, J., Hsu, C.-S., Bai, L., Chen, H.M. & Hu, X. Atomically dispersed Fe3+ sites catalyze efficient CO2 electroreduction to CO. **364**, 1091-1094 (2019).
25. Yang, H.B. et al. Atomically dispersed Ni(i) as the active site for electrochemical CO2 reduction. *Nature Energy* **3**, 140-147 (2018).
26. Pei, J. et al. A replacement strategy for regulating local environment of single-atom Co-SxN4−x catalysts to facilitate CO2 electroreduction. *Nature Communications* **15**, 416 (2024).
27. Jiao, J. et al. Constructing asymmetric double-atomic sites for synergistic catalysis of electrochemical CO2 reduction. *Nature Communications* **14**, 6164 (2023).
28. Wang, Q. et al. Attenuating metal-substrate conjugation in atomically dispersed nickel catalysts for electroreduction of CO2 to CO. *Nature Communications* **13**, 6082 (2022).
29. Zeng, Z. et al. Orbital coupling of hetero-diatomic nickel-iron site for bifunctional electrocatalysis of CO2 reduction and oxygen evolution. *Nature Communications* **12**, 4088 (2021).
30. Hossain, M.D., Huang, Y., Yu, T.H., Goddard Iii, W.A. & Luo, Z. Reaction mechanism and kinetics for CO2 reduction on nickel single atom catalysts from quantum mechanics. *Nature Communications* **11**, 2256 (2020).
31. Zhang, B. et al. Manganese acting as a high-performance heterogeneous electrocatalyst in carbon dioxide reduction. *Nature Communications* **10**, 2980 (2019).
32. Zheng, T. et al. Large-Scale and Highly Selective CO2 Electrocatalytic Reduction on Nickel Single-Atom Catalyst. *Joule* **3**, 265-278 (2019).
