# Supplementary material for: Lanthanide single-atom catalysts for efficient CO2-to-CO electroreduction
Source: Nat Commun. 2025 Mar 27;16:2985. doi: 10.1038/s41467-025-57464-8 (PMC11947204; doi:10.1038/s41467-025-57464-8)
Supplement: Supplementary file 2 — Description Of Additional Supplementary File [file 41467_2025_57464_MOESM2_ESM.pdf]

Description of Additional supplementary file

Supplementary Data 1:

Description: The DFT-optimized computational models for the CO_2 reduction to CO on Er single atom catalyst, Ca single atom catalyst and Fe single atom catalyst.
